# Topological Reconfiguration in Sheared Granular Assemblies: Non-Dissipative Mechanism and Its Irreversible Characteristics

**DOI:** 10.3390/ma19204291

**Published:** 2026-10-12

**Authors:** Xiaoxiao Wang, Peng Li, Pengqiang Yu, Yang Liu

**Affiliations:** 1 Department of Civil Engineering, University of Science and Technology Beijing, Beijing 100083, China; xiaoxiao.wang@ustb.edu.cn (X.W.); pq_yu@ustb.edu.cn (P.Y.); 2 Sichuan Provincial Airfield Engineering Co., Ltd., Chengdu 610084, China; lp28537841@163.com

**Keywords:** granular materials, topological reconfiguration, elastic energy storage and release, irreversible free energy, mesoscopic loops

## Abstract

Granular materials exhibit pronounced macroscopic irreversible deformations during shearing, which mainly contributes to frictional sliding dissipation in the conventional thermodynamic frameworks. This study aims to investigate the non-dissipative microscopic mechanisms governing macroscopic irreversibility of granular materials. Using DEM, a series of biaxial shear and reverse loading simulations are conducted on dense granular assemblies with different friction coefficients. The results show that continuous topological reconfiguration occurs through the transformation from lower-order to higher-order meso-loops, eventually reaching a statistical steady state at the critical state. Accompanying this topological evolution, elastic energy at contact level is continuously stored and released, providing a persistent non-dissipative energy exchange that sustains continuous shear alongside primary frictional dissipation. Based on the reverse loading analysis, topological reconfiguration is intrinsically irrecoverable, causing part of the free energy to remain locked within the reconfigured contact network as irrecoverable free energy. Meanwhile, another portion of the recoverable free energy is irreversibly dissipated through friction during unloading. Together, these two components constitute the irreversible free energy. The evolution of irreversible free energy with interparticle friction is consistent with those of the critical frictional dissipation and macroscopic volumetric strain, indicating that the macroscopic irreversible deformation of granular assemblies is governed not only by frictional dissipation but also by the irreversible evolution of free energy.

## 1. Introduction

Geomaterials are typical heterogeneous granular assemblies whose complex macroscopic mechanical behavior results from the continuous evolution of the internal contact network. The elastic response of granular materials depends strongly on the current contact configuration, whereas the evolution of this configuration is governed by irreversible particle rearrangements during deformation [1,2,3]. In this sense, it is inappropriate to explain the distinction between reversible and irreversible deformation of granular materials within the framework of classical elastoplasticity. Instead, it should also account for the evolution of the internal contact topology and its associated structural reconfiguration.

Over the past decades, the micromechanical mechanisms of the macroscopic behavior of granular materials have been extensively investigated through experimental techniques, such as photoelasticity and X-ray computed tomography [4,5,6], together with the discrete element method (DEM) [7,8,9]. At the microscopic scale referring to the particle and contact level, many studies [10,11,12,13,14] have demonstrated that contact sliding and the associated frictional dissipation are the primary sources of macroscopic plastic deformation, and are closely related to complex mechanical behavior including strain localization, strain softening, dilatancy, and the critical state. Accordingly, most existing micromechanical theories [15,16,17] establish the link between microscopic mechanisms and macroscopic responses from the perspective of frictional dissipation, which is generally regarded as the dominant mechanism governing irreversible deformation. However, recent studies [18] in granular physics have shown that irreversible shear deformation can still develop in frictionless granular assemblies during the jamming–unjamming transition. This finding suggests the existence of an additional irreversible mechanism independent of frictional dissipation [19]. Pouragha et al. [20] classified the micromechanical mechanisms of granular materials into three categories: (i) a dissipative irreversible mechanism associated with contact sliding; (ii) a non-dissipative reversible mechanism associated with elastic contact deformation; and (iii) a non-dissipative irreversible mechanism arising from contact creation, contact loss, and the resulting topological evolution of the contact network. Some studies [21,22,23,24] further demonstrated that topological evolution induces particle rearrangement, fabric anisotropy, and microstructural evolution, thereby playing an important role in heterogeneous deformation [25,26,27]. Nevertheless, current studies on the non-dissipative irreversible mechanism remain focused on statistical descriptions of structural evolution, and its fundamental contribution to macroscopic irreversible deformation has not yet been clearly established.

As a fundamental topological unit of the contact network, the mesoscopic loop structure enclosed by minimum number of particles in contact provides a proper framework for characterizing the structural evolution of granular assemblies. Recently researchers [28,29,30,31] have devoted themselves to investigating the geometric and mechanical characteristics of loop structures and their relationship with the critical state. Previous studies [32,33] showed that the macroscopic critical state corresponds to the statistical stabilization of the geometric and mechanical characteristics of different loop types. On this basis, the mesoscopic critical state line was proposed to establish the cross-scale correspondence between mesoscopic topology and the macroscopic critical state [34]. Beyond static statistical analyses, Deng et al. [35] showed that the critical state is characterized by a dynamic equilibrium between contact creation and contact loss, which leads to the continuous generation and disappearance of loop structures. Sun et al. [36] further reported that loop reconfiguration competes with frictional dissipation. Nicot et al. [37] introduced the concept of configurational entropy to describe this structural evolution. Wang et al. [38] showed that configurational evolution is one of the primary origins of fluctuations in macroscopic mechanical responses and should therefore be incorporated into constitutive modeling. These studies indicate that revealing the transient reconfiguration and irrecoverability of loop structures during shearing is essential for understanding the contribution of non-dissipative mechanisms to irreversible deformation.

From a thermodynamic perspective, under quasi-static loading, the external work input into a granular assembly is partitioned into two parts: one is dissipated through contact sliding, while the other is stored within particle contacts as elastic energy. Existing studies on energy evolution have primarily focused on frictional dissipation [12,14], whereas the role of the non-dissipative elastic energy process remains less understood [39]. During topological reconfiguration, contact creation stores elastic strain energy, whereas contact loss results in elastic energy release, giving rise to a continuous dynamic elastic energy exchange throughout shearing. Previous studies [38,40] have shown that this process persists during deformation and reaches a statistical balance at the critical state. Regarding the non-dissipative irreversibility mechanism, Collins [41] introduced the concept of frozen free energy and explained it as the energy trapped within compressed structural networks. Nicot and Darve [2] proposed that only part of the free energy stored during loading can be recovered upon unloading, and introduced the concept of configuration energy [42]. More recently, Liu and Wang [43] identified the irreversible component of free energy and established a stress–dilatancy relationship incorporating irreversible free energy. However, two key research issues are still open: (i) the physical link between meso-loop reconfiguration and the irreversibility of free energy has not yet been quantitatively established; (ii) the respective energy contributions of non-dissipative structural reconfiguration and secondary frictional dissipation during recovery have not been clearly distinguished.

To address these issues, this study investigates the topological reconfiguration and its irreversible characteristics within granular assemblies, with particular emphasis on the non-dissipative microscopic mechanisms associated with macroscopic irreversible deformation. Based on a series of DEM biaxial shear simulations of dense granular assemblies with different friction coefficients, this study first identifies persistent meso-loop reconfiguration as a non-dissipative microscopic mechanism accompanying sustained irreversible deformation. The associated elastic energy storage and release are quantified, revealing a statistically steady exchange at the critical state despite continued microscopic reorganization. Reverse loading results then demonstrate that macroscopic stress recovery does not imply recovery of the contact network topology, and that a finite residual elastic energy remains after stress recovery. This residual energy is used to quantify the irrecoverable free energy component associated with topological reconfiguration, while frictional dissipation during reverse loading quantifies the irreversible loss of the recoverable component. By combining these two contributions, an irreversible free energy established to provide an energetic description of irreversible deformation beyond frictional dissipation alone.

The remainder of this paper is organized as follows. First, a series of DEM biaxial simulations on dense granular assemblies with different interparticle friction coefficients are performed in Section 2. Then, the topological reconfiguration of meso-loops during shearing is characterized, together with the non-dissipative mechanism from the perspective of elastic energy storage and release at contacts in Section 3. Finally, reverse loading simulations are conducted in Section 4 to examine the irrecoverability of both topological reconfiguration and free energy, providing a physical interpretation of the non-dissipative mechanisms governing macroscopic irreversible shear deformation in granular assemblies.

## 2. DEM Simulations

### 2.1. DEM Setup for Biaxial Loading Tests

In this study, two-dimensional (2D) drained biaxial loading simulations of granular assemblies are conducted using the PFC2D version 5.0 (Particle Flow Code in 2 Dimensions) software based on the Discrete Element Method (DEM) [44]. The use of a 2D granular assembly is motivated by the topological focus of the present study. In particular, the meso-loops used to characterize the topology of the contact network can be explicitly identified and tracked in a two-dimensional contact network, allowing their generation, disappearance, and reconfiguration to be quantified directly.

**Figure 1:**
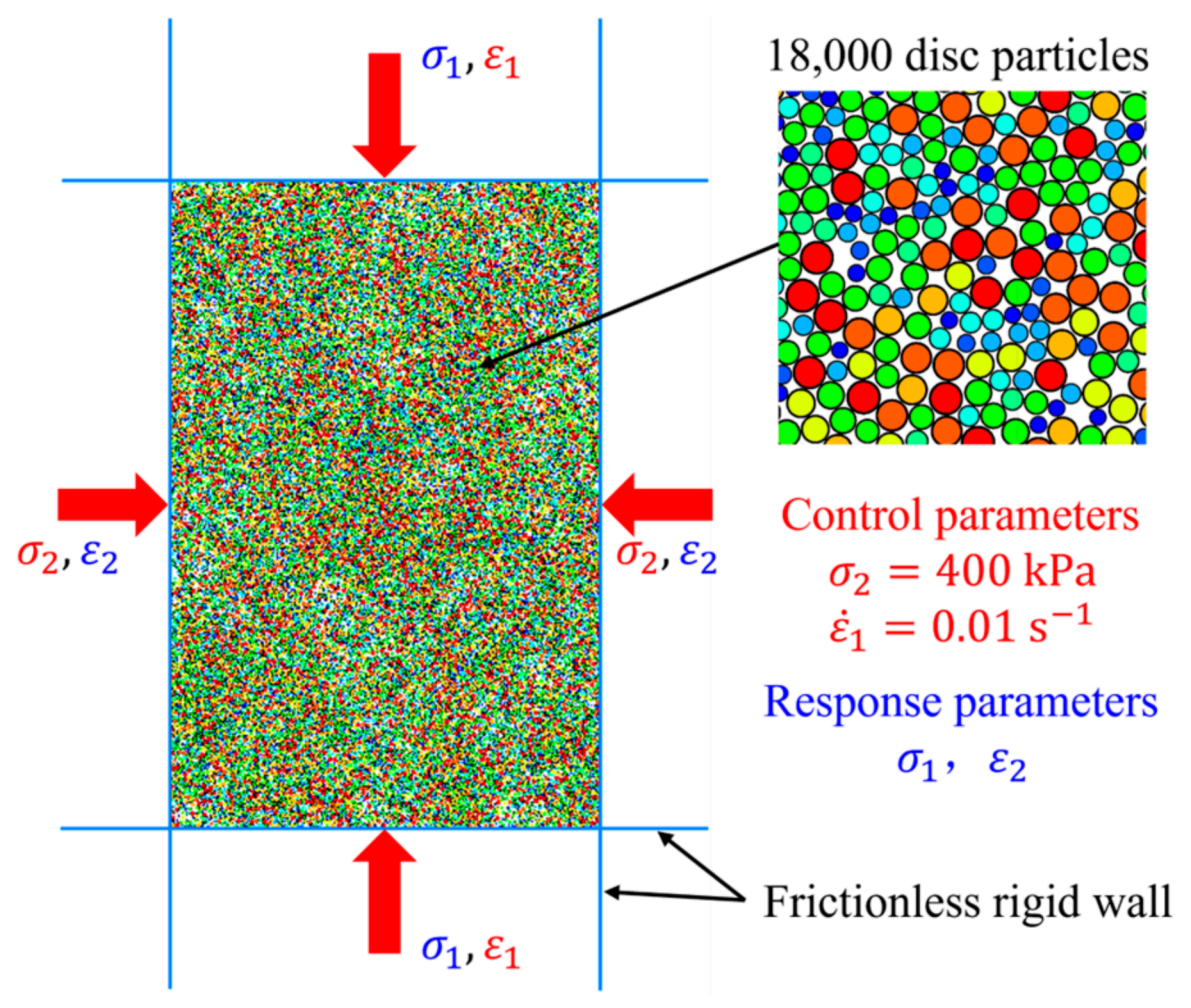
Schematic diagram of the 2D DEM biaxial test model and loading conditions. Different particle colors represent different particle sizes.

A granular assembly containing 18,000 particles is randomly generated within a rectangular domain with a width of 1.0 m and a height of 1.5 m, as shown in Figure 1. The specimen is enclosed by frictionless rigid walls to eliminate boundary friction effects during specimen preparation and loading. The particle radius is uniformly distributed within the range of 3–6 mm, with an average particle diameter *D*_50_ of 0.009 m. The particle density is set to 2600 kg/m^3^. A linear contact model is adopted to describe the interparticle interactions. For a contacting particle pair, the normal and tangential contact forces *F_n_* and *F_t_* are calculated from the relative displacements,

(1) $$\left\{\begin{array}{c} F_{n}=k_{n}u_{n}\\ dF_{t}=k_{t}du_{t},\begin{array}{c} F_{t}\leq \mu F_{n} \end{array} \end{array}\right.$$

where *k_n_* and *k_t_* denote the normal and tangential stiffness, respectively; *u_n_* is the total normal relative displacement; d*u_t_* is the incremental tangential relative displacement; and *μ* is the interparticle friction coefficient. When the tangential force reaches the frictional limit, sliding occurs and the magnitude of the tangential force is constrained by *F_t_* = *μF_n_*. In the present simulations, the normal and tangential contact stiffnesses are both set to 5 × 10^8^ N/m, ensuing that the maximum relative contact overlap remains well below 0.1% throughout the shearing process. During specimen generation, the interparticle friction coefficient *μ* is set to zero to facilitate particle rearrangement and obtain a highly compact initial configuration.

After specimen generation, an isotropic confining pressure of 400 kPa is applied to consolidate the assembly until an isotropic stress state is reached. The final porosity of the consolidated specimen is 0.156, corresponding to a relatively dense state. Subsequently, drained biaxial loading tests are conducted under a constant confining pressure of 400 kPa. During this shearing stage, the top and bottom walls are displaced in opposite directions at a constant axial strain rate ${\dot{\varepsilon }}_{1}$ of 0.01 s^−1^, while the lateral walls are servo-controlled to maintain the prescribed confining pressure *σ*_2_. The corresponding boundary conditions can therefore be summarized as

(2) $$\left\{\begin{array}{c} \sigma_{2}=400\mathrm{kPa}\\ {\dot{\varepsilon }}_{1}=0.01^{-1} \end{array}\right.$$

To investigate the non-dissipative microscopic mechanisms governing the macroscopic irreversibility of granular materials, the consolidated dense specimen is replicated to initiate seven series of biaxial shearing simulations. At the onset of shearing, the interparticle friction coefficient is reset to *μ* = 0.01, 0.1, 0.2, 0.3, 0.4, 0.5, and 0.75, respectively. Except for the friction coefficient *μ*, all other contact and physical parameters during the shearing stage are kept strictly identical to those used during the consolidation stage. It should be noted that varying *μ* modifies both the frictional resistance and the extent of interparticle sliding, thereby affecting frictional dissipation as well as contact network reconfiguration. The initial particle arrangement may affect the transient mechanical response and local evolution of the contact network during subsequent shearing. Using the same initial configuration for all simulations removes this source of variability and allows the effects of the shearing friction coefficient to be examined under otherwise identical initial conditions. This choice is intended to provide a controlled comparison rather than to assume that the initial arrangement has no influence. A systematic assessment based on multiple independently generated initial configurations is beyond the scope of the present study and is recognized as a limitation of the current numerical framework. A very small damping coefficient of 0.01 is adopted to suppress numerical fluctuation while minimizing artificial damping dissipation. Together with the low loading rate, this treatment ensures that the simulations remain close to the quasi-static regime. Therefore, this controlled numerical framework provides a basis for examining the coupled evolution of frictional dissipation, topological reconfiguration, and macroscopic irreversible deformation.

### 2.2. Macroscopic Responses

**Figure 2:**
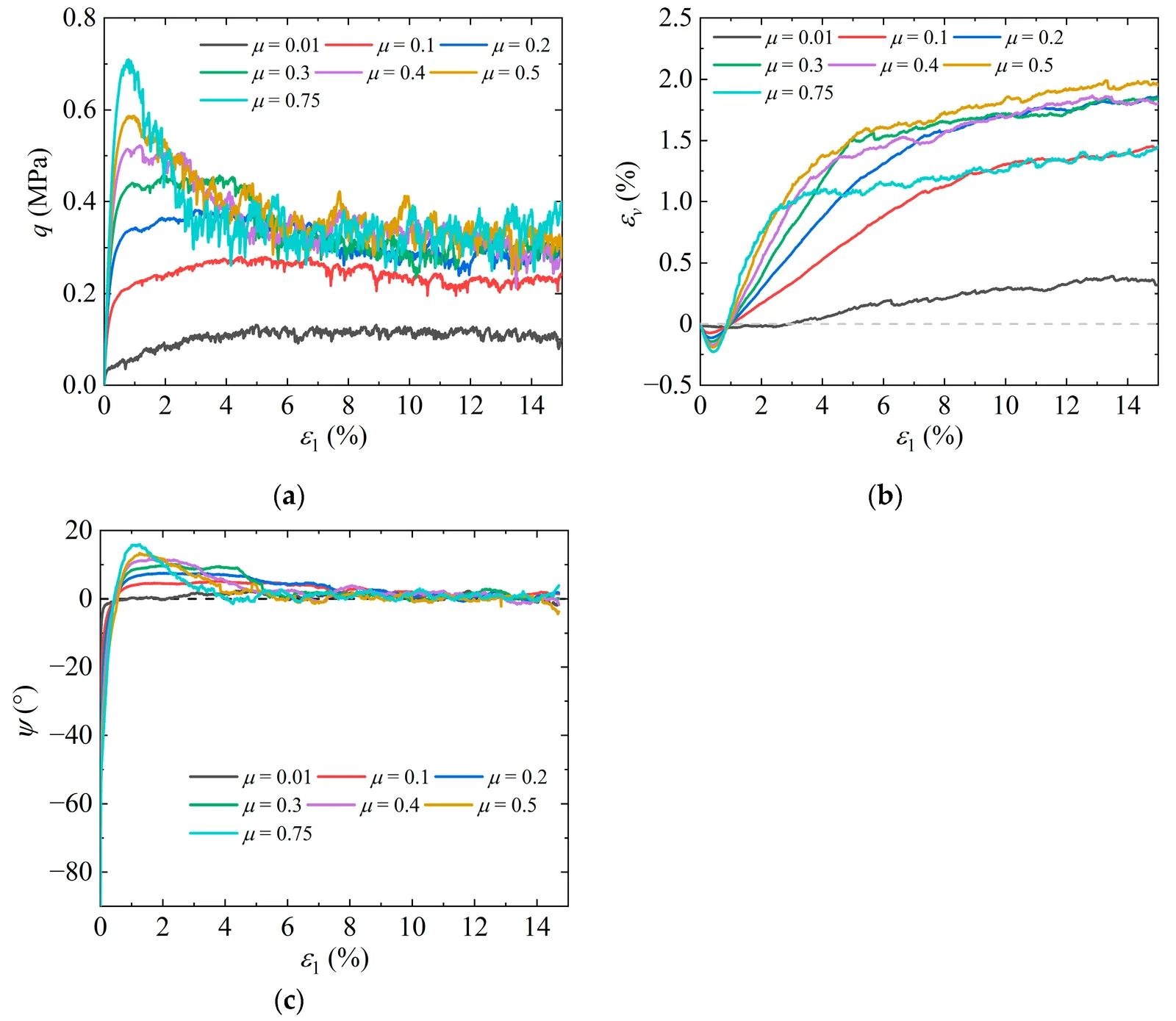
Evolutions of (**a**) deviatoric stress *q*, (**b**) volumetric strain *ε*_v_ and (**c**) dilatancy angle *ψ* with axial strain *ε*_1_ under different frictional conditions.

Figure 2 illustrates the macroscopic mechanical responses obtained from the 2D DEM biaxial simulations for the dense specimens under different frictional conditions. The responses are characterized by the evolutions of deviatoric stress *q*, volumetric strain *ε*_v_, and dilatancy angle *ψ* with axial strain *ε*_1_. For biaxial loading, these macroscopic quantities are calculated as

(3) $$\begin{array}{c} q=\sigma_{1}-\sigma_{2}\\ \varepsilon_{v}=\varepsilon_{1}+\varepsilon_{2}\\ \sin\psi =-\frac{d\varepsilon_{v}}{d\varepsilon_{\gamma }}=-\frac{d\varepsilon_{1}+d\varepsilon_{2}}{d\varepsilon_{1}-d\varepsilon_{2}} \end{array}$$

where *σ*_1_ and *σ*_2_ denote the axial and lateral stresses, respectively; *ε*_1_ and *ε*_2_ represent the axial and lateral strains, respectively; and *ε_γ_* denotes the shear strain. The sign convention adopted in the DEM simulations takes compression as negative and extension as positive. Note that the horizontal axes in all figures in this study showing the evolution of response variables with axial strain are plotted against the absolute value of the axial strain *ε*_1_, such that the applied compressive deformation increases from left to right.

As shown in Figure 2a, all specimens exhibit the typical shear response of dense granular materials under different frictional conditions. The deviatoric stress initially increases to a peak value, followed by pronounced post-peak strain softening, and eventually approaches a stable residual state at large strain, corresponding to the critical state. For the lowest friction condition (*μ* = 0.01), both the peak strength and post-peak softening are relatively limited. As *μ* increases, the peak deviatoric stress gradually increases, while the corresponding axial strain decreases, indicating an earlier transition to the post-peak softening regime. At the critical state, the residual deviatoric stress generally increases with increasing *μ*. However, when *μ* > 0.3, the critical deviatoric stress becomes less sensitive to further increases in the friction coefficient.

Regarding the volumetric strain response in Figure 2b, all specimens initially exhibit contractancy followed by dilatancy, and eventually approach a stable volumetric strain at large strain. As *μ* increases, the maximum volumetric contraction increases, accompanied by an increase in the critical volumetric strain. Similarly, when 0.3 < *μ* ≤ 0.5, the critical volumetric response becomes progressively less sensitive to further increases in friction. Notably, when *μ* is further increased to 0.75, the critical volumetric strain decreases compared to that at *μ* = 0.5. The microscopic mechanisms underlying this non-monotonic frictional effect on macroscopic behavior will be further investigated in the following sections.

The evolution of the dilatancy angle *ψ* is shown in Figure 2c. The value of *ψ* is negative during the initial loading stage, indicating contractive behavior for all friction coefficients. As shearing continues, *ψ* increases toward zero, marking the transition from contractancy to dilatancy, and subsequently reaches a positive peak. Note that higher interparticle friction coefficients are associated with larger peak dilatancy angles. With further shearing, *ψ* decreases and fluctuates around zero, suggesting that the incremental volumetric strain becomes nearly zero as the granular assembly approaches the critical state, where sustained shear deformation occurs at an approximately constant volume.

Based on the macroscopic mechanical responses, three representative characteristic states are selected for the subsequent analysis, namely the maximum contraction state A, peak stress state B, and critical state C. The axial strains corresponding to the three characteristic states under different friction coefficients are summarized in Table 1. Note that, since the axial strain corresponding to the critical state varies slightly under different frictional conditions, a unified axial strain of 12% is considered as the representative critical state C.

### 2.3. Frictional Dissipation

**Figure 3:**
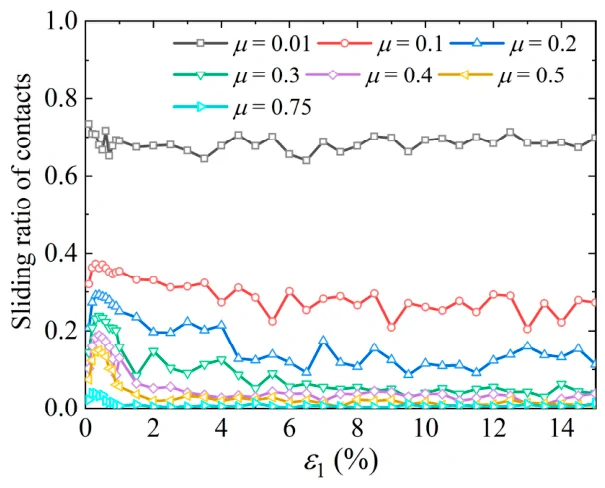
Evolution of the sliding contact ratio during biaxial shearing under different frictional conditions.

The evolution of the sliding contact ratio during biaxial shearing under different frictional conditions is presented in Figure 3. It can be observed that the sliding contact ratio initially increases slightly, then gradually decreases, and eventually approaches a nearly constant value at the critical state. For the lowest friction condition (*μ* = 0.01), approximately 70% of the contacts remain in a sliding state throughout the shearing process due to the weak tangential frictional resistance between particles. As *μ* increases, the sliding contact ratio decreases. When *μ* > 0.3, it becomes less sensitive to further increases in friction and approaches zero at the critical state, indicating that active contact sliding becomes increasingly limited under high-friction conditions.

During shearing, external work is continuously inputted into the granular assembly, part of which is dissipated through relative sliding at interparticle contacts. The calculation of the cumulative frictional dissipation energy *E*^fric^ in granular systems is detailed in Wang et al. (2024) [40]. Due to the persistent occurrence of contact sliding (in Figure 3), frictional dissipation energy continuously accumulates. To characterize the rate of energy dissipation under different frictional conditions, the frictional dissipation power *P*^fric^ is defined as

(4) $$P^{\mathrm{fric}}=\frac{dE^{\mathrm{fric}}}{dt}$$

where d*t* represents the computational time increment during an incremental loading step considered. To eliminate the influence of specimen size variation, the frictional dissipation power is normalized by the current specimen volume. In addition, because the instantaneous particle rearrangement occurring within the granular assembly during shearing may induce high frequency fluctuations in the energy response, a moving average smoothing procedure based on adjacent data points is applied to the original curves to obtain clearer evolution trends.

**Figure 4:**
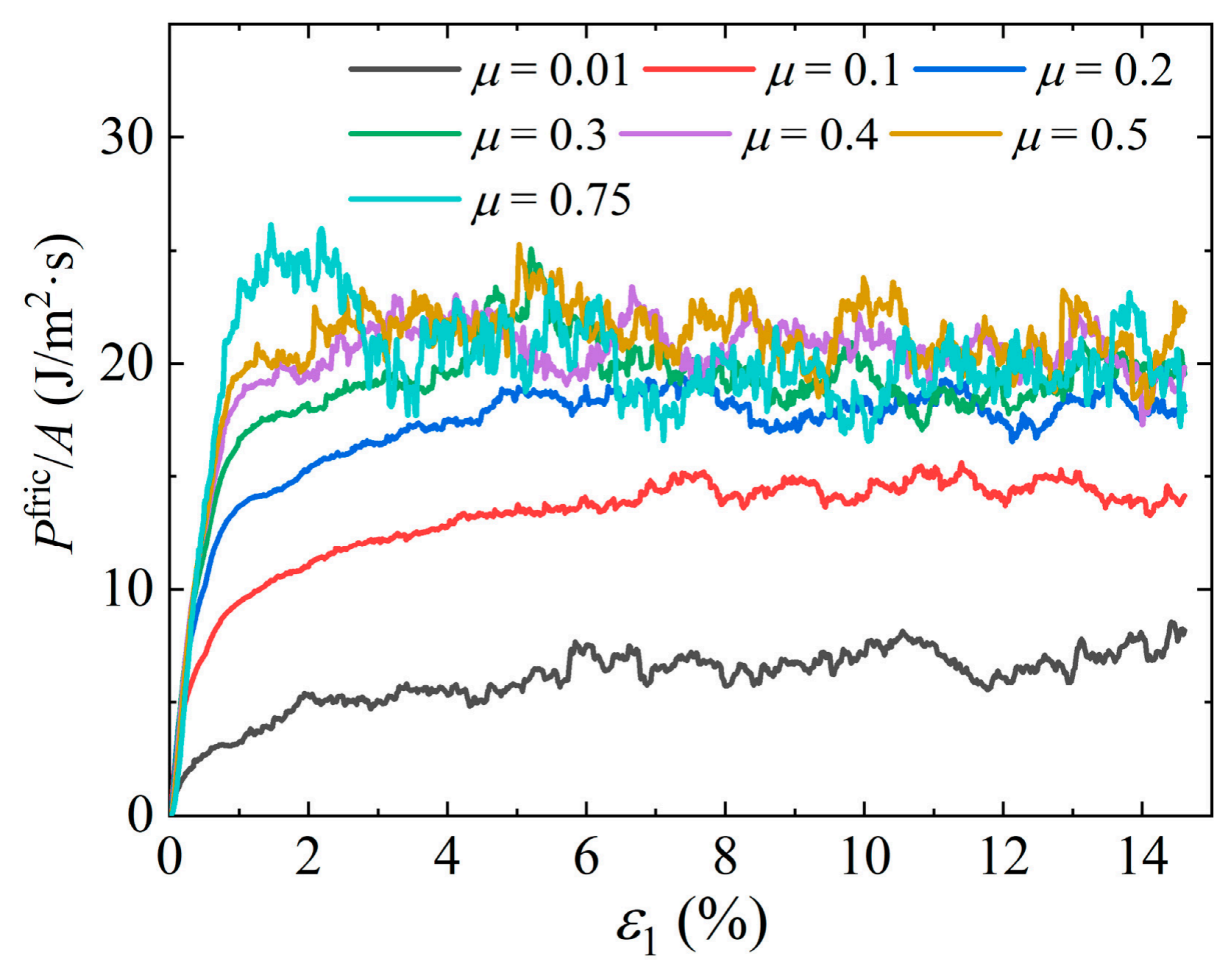
Evolution of the frictional dissipation power during biaxial shearing under different frictional conditions, normalized by current specimen volume *A*.

Figure 4 presents the evolution of the normalized frictional dissipation power *P*^fric^ with axial strain under different frictional conditions. For the lowest friction condition (*μ* = 0.01), *P*^fric^ increases gradually from an initial value close to zero and eventually approaches a stable level at large strain. As *μ* increases, the initial increase rate of *P*^fric^ becomes significantly steeper, reflecting the combined effects of increased tangential resistance and changes in contact-sliding activity. With continued shearing, *P*^fric^ reaches a maximum at a certain shear state, followed by a gradual decrease associated with the progressive structural reorganization of the granular assembly, and finally approaches a stable level at large strain.

**Figure 5:**
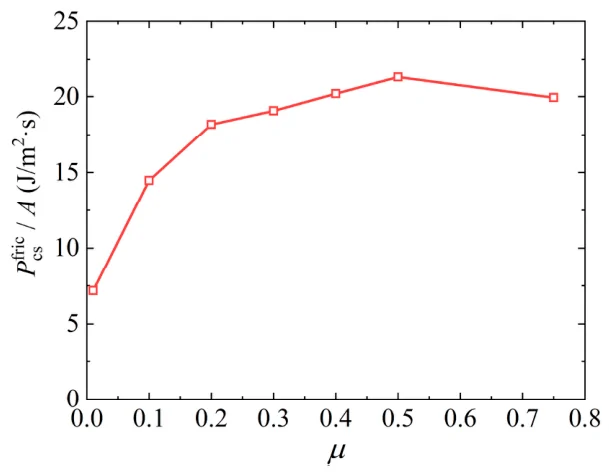
Variation in normalized frictional dissipation power at the critical state in terms of the friction coefficient.

Figure 5 further illustrates the variation in normalized frictional dissipation power at the critical state in terms of the friction coefficient *μ*. Here, the average value within the axial strain range of 12–14% is represented as the critical dissipation power. It can be observed that the critical frictional dissipation power increases nonlinearly with increasing *μ*, with a pronounced increase in the low-friction regime and a progressively weaker increase at higher friction coefficients. Among the investigated cases, the dissipation power reaches its maximum at *μ* = 0.5 and decreases slightly at *μ* = 0.75, indicating that the effect of increasing friction on macroscopic frictional dissipation is not strictly monotonic over the investigated range. At the contact scale, frictional energy dissipation rate depends on both the contact tangential forces and the relative sliding velocities. Increasing *μ* raises the frictional resistance and can therefore increase the energy dissipated during individual sliding events, while higher *μ* suppresses relative sliding, thereby reducing the number of actively sliding contacts. At low to intermediate friction, the increase in frictional resistance dominates, leading to a higher dissipation power. At higher friction, the reduction in sliding activity increasingly offsets the increase in frictional resistance, resulting in the slight decrease observed at *μ* = 0.75.

## 3. Non-Dissipative Mechanisms Within Granular Assemblies

With continuous input of external work, part of the energy is dissipated through interparticle friction, while another part is stored temporarily as elastic energy at particle contacts. As presented above, both the frictional dissipation power and the volumetric strain at the critical state decrease when the friction coefficient exceeds 0.5, suggesting that the underlying deformation mechanism changes under high-friction conditions. In this sense, it motivates a further investigation into the non-dissipative mechanisms associated with topological reconfiguration and elastic energy processes.

The analysis presented in this section is based on two underlying assumptions. First, the internal structure of the two-dimensional granular assembly is represented as a planar contact network, in which particles and interparticle contacts constitute the nodes and edges, respectively. The bounded faces of this network are regarded as mesoscopic loop structures, and topological reconfiguration is identified through changes in contact connectivity, including contact formation and disappearance and the resulting generation, disappearance, and transformation of meso-loops. Accordingly, particle displacement and topological reconfiguration are treated as distinct but related aspects of the granular response. Second, the proposed topological and energetic description is established within the linear elastic-frictional contact framework adopted in the DEM simulations. The elastic component of the contact law governs the storage and release of elastic energy, whereas interparticle friction provides the dissipative contribution associated with relative sliding. Thus, the non-dissipative mechanism discussed here does not constitute an additional constitutive law or an independent energy source, but rather describes the topological reconfiguration and elastic energy exchange that emerge from the underlying contact interactions.

### 3.1. Topological Reconfiguration

**Figure 6:**
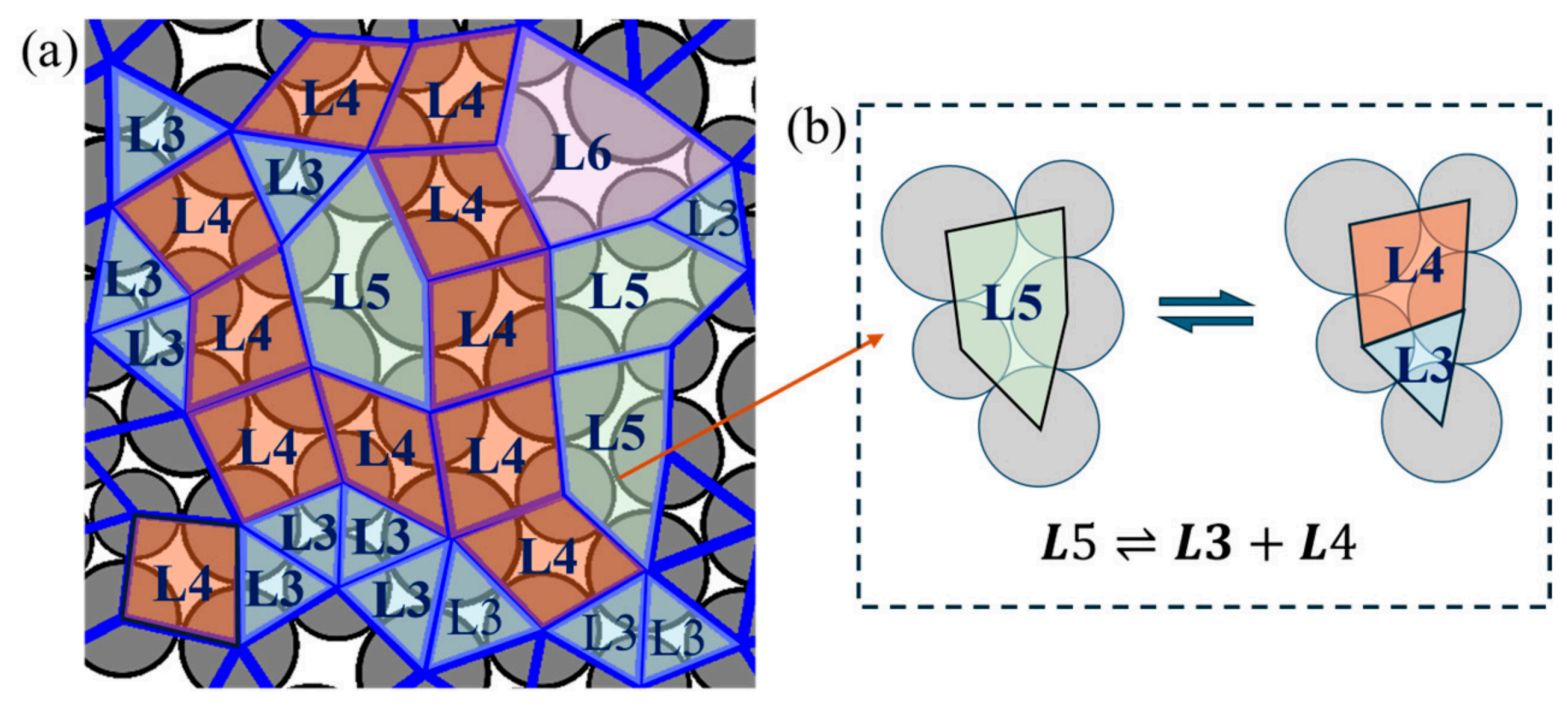
Schematic diagram of (**a**) the meso-loop tessellation within a 2D granular medium and (**b**) an example of the configuration transformations of meso-loops. Different fill colors distinguish meso-loops of different orders (L3, L4, L5, and L6), while blue lines represent interparticle contacts.

To characterize the evolution of the internal contact network from a topological perspective, the granular assembly is represented as a planar contact graph (*G* = (*P*,*C*)), where *P* denotes the set of particles and *C* denotes the set of interparticle contacts. The smallest enclosed regions bounded by the contact network, i.e., the bounded faces of the planar contact graph, are adopted as the fundamental topological units and referred to as mesoscopic loop structures, as illustrated in Figure 6a. Each loop is therefore uniquely defined by a closed sequence of contacting particle pairs. The loops are identified from the contact graph by tracing connected contact edges around the enclosed regions, with each bounded face counted once. The order of a loop, denoted by *n*, is defined as the number of contact edges forming its closed boundary. Accordingly, the loop structures are classified into different categories L*n*, i.e., L3, L4, L5, and L6+, where L6+ includes these loops with six or more orders.

The loop-based description provides information that is fundamentally different from particle displacement. The evolution of particle positions describes the kinematic motion of individual particles, whereas changes in the set of interparticle contacts represent reconfiguration of the contact network topology. Particles may undergo finite displacements while retaining the same contact connectivity, in which case the network topology remains unchanged. Conversely, the formation or disappearance of only a small number of contacts can change the topology even when the associated particle displacements are relatively small. Therefore, the meso-loop representation provides a structural description that directly quantifies how particle connectivity and the organization of void regions evolve during shearing.

As illustrated in Figure 6b, a meso-loop L5 is subdivided into two lower-order loops L3 and L4 due to the formation of a new contact. Conversely, the disappearance of an existing contact reverses this transformation. Therefore, the generation and disappearance of meso-loops represent continuous reorganization of the contact network and provide a direct manifestation of topological reconfiguration. To quantitatively characterize this process, the generation ratio $R_{Ln}^{+}$ and disappearance ratio $R_{Ln}^{-}$ of meso-loops are defined as the ratios of the numbers of newly generated and disappeared loops of a given type, respectively, to the total number of contacts within a given incremental loading step, including both unchanged and newly evolved contacts. Previous studies [29,45] have shown that the dominant topological evolution during shearing is characterized by the transformation of lower-order L3 into higher-order L6+. In this sense, Figure 6 shows the generation and disappearance ratios of L3 and L6+ during shearing, after normalized by the incremental axial strain of d*ε*_1_ = 0.1% considered.

**Figure 7:**
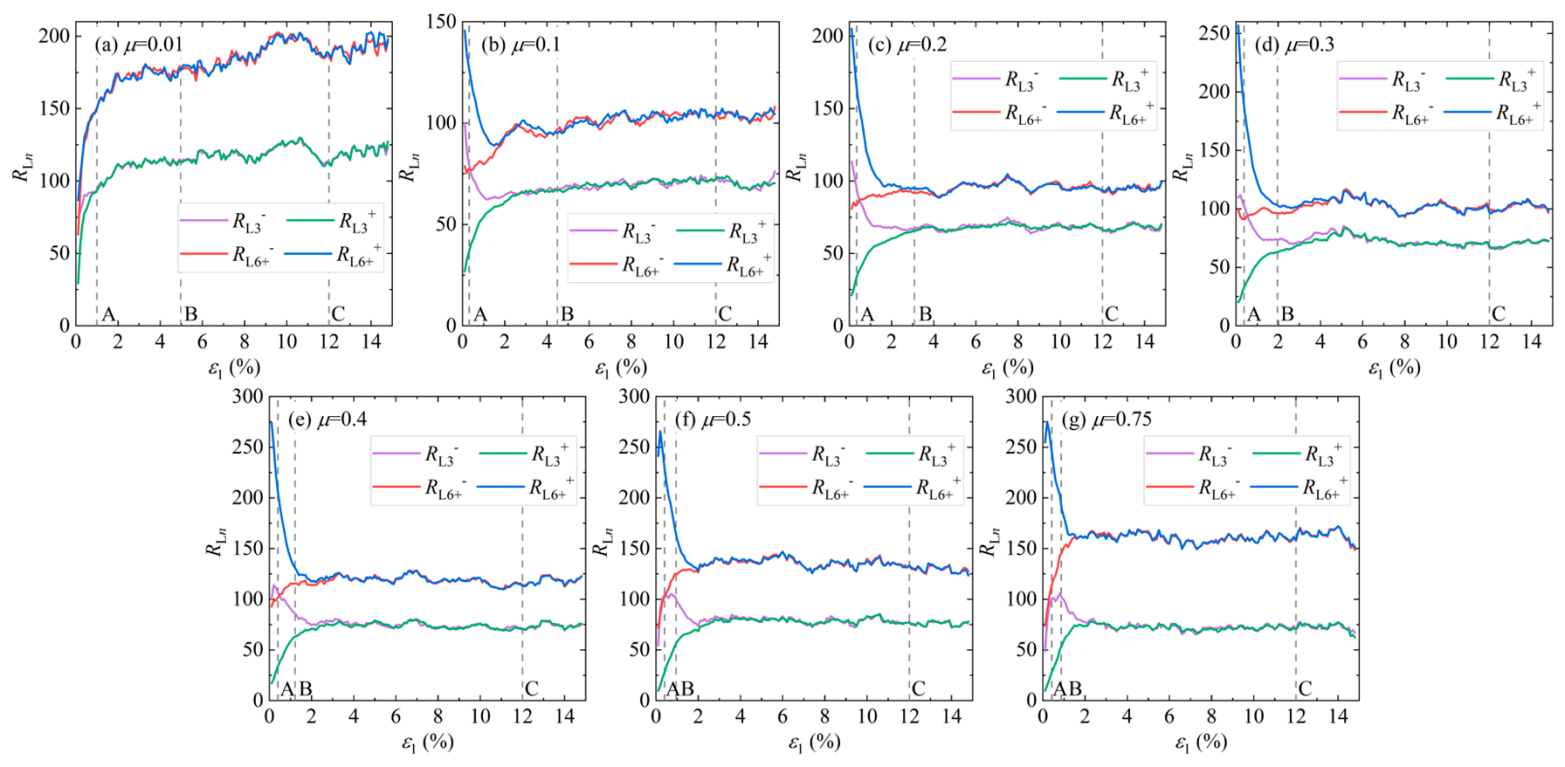
Generation and disappearance ratios of L3 and L6+ loops during shearing, after normalized by the incremental axial strain of d*ε*_1_ = 0.1% considered, under different interparticle friction coefficients: (**a**) *μ* = 0.01; (**b**) *μ* = 0.1; (**c**) *μ* = 0.2; (**d**) *μ* = 0.3; (**e**) *μ* = 0.4; (**f**) *μ* = 0.5; and (**g**) *μ* = 0.75. The vertical dashed lines indicate three characteristic states: maximum contraction state A, peak stress state B, and critical state C, with their corresponding axial strains listed in Table 1.

As shown in Figure 7, for all frictional conditions, the disappearance ratio $R_{L3}^{-}$ of L3 is larger than their generation ratio $R_{L3}^{+}$ during the initial loading stage, whereas the opposite trend is observed for L6+. This suggests that the contact network continuously evolves from lower-order to higher-order loops during the initial loading stage. This imbalance is relatively weak under the lowest friction condition (*μ* = 0.01) but becomes much more pronounced at higher friction coefficients, indicating that stronger interparticle friction promotes sustained topological evolution associated with particle rearrangement. As shearing proceeds, the generation and disappearance ratios of both L3 and L6+ gradually converge after the peak stress state (State B) and eventually stabilize at nearly identical values. This suggests that the rates of loop generation and disappearance become statistically balanced during continued quasi-static shearing. Consequently, the overall topological composition remains invariant in a statistical sense, which directly corresponds to the macroscopic critical state behavior characterized by continuous deformation at constant volume and stress.

### 3.2. Elastic Energy Evolution

**Figure 8:**
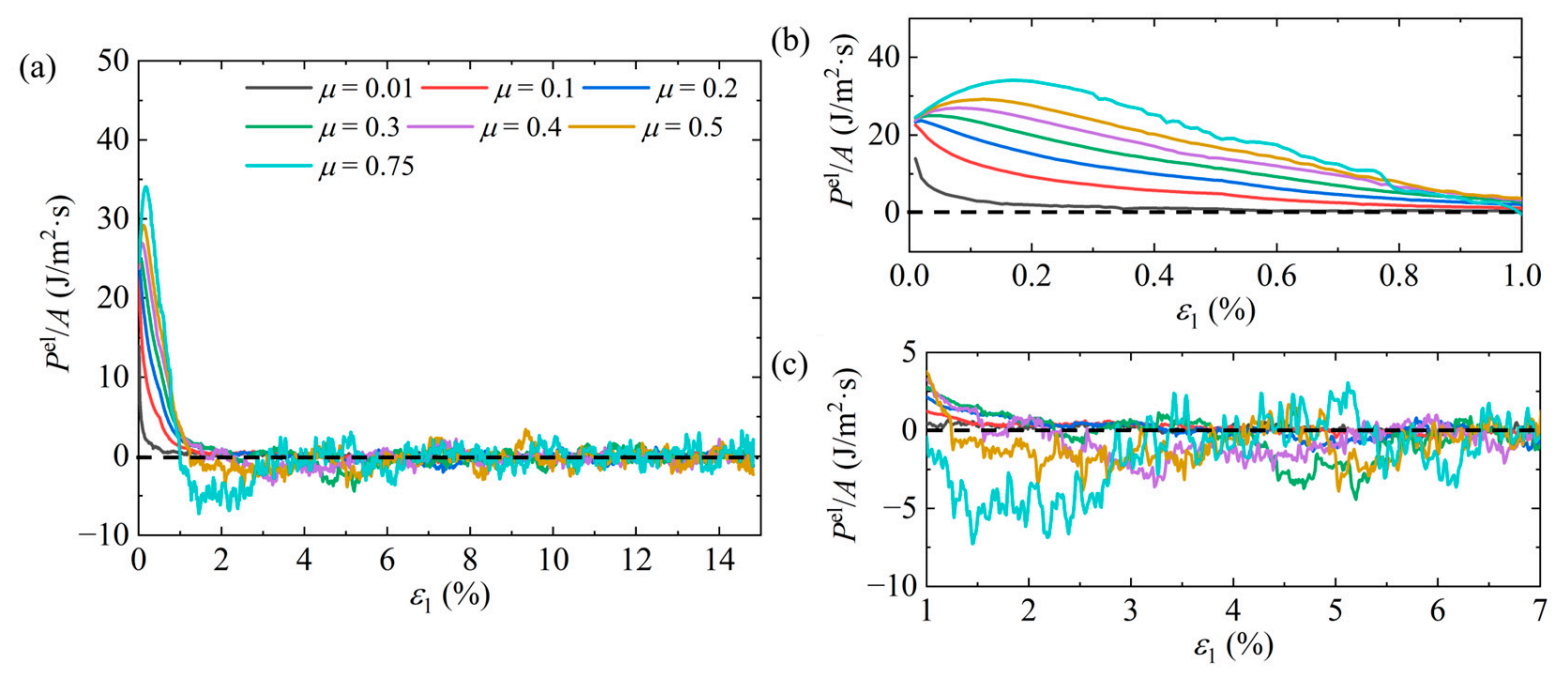
Evolution of the normalized elastic power under different frictional conditions: (**a**) entire shearing process; (**b**) enlarged view of the initial loading stage; (**c**) enlarged view of the post-peak softening stage. The horizontal dashed lines indicate the zero reference level of the net elastic energy rate.

The calculation of elastic strain energy stored at an individual contact within a granular assembly follows the method detailed in Wang et al. (2024) [40]. At any mechanical state, the total elastic energy stored in the granular assembly *E*^el^ is obtained by summing the elastic energy over all contacts. To characterize the evolution of elastic energy during shearing, the elastic power is defined as *P*^el^ = d*E*^el^/d*t*. Figure 8 presents the evolution of *P*^el^ under different frictional conditions, normalized by the specimen volume *A*. Note that the curves are smoothed using a moving average smoothing procedure based on adjacent data points. Figure 8b,c provide enlarged views of the initial loading stage and the post-peak softening stage, respectively, to highlight the detailed evolution of the elastic power.

During the initial loading stage, *P*^el^ remains positive, indicating a continuous increase in the total elastic energy stored within the granular assembly. This corresponds to the progressive increase in the macroscopic deviatoric stress. Under low-friction conditions (*μ* < 0.3), *P*^el^ decreases gradually from an initial positive value. When *μ* ≥ 0.3, *P*^el^ exhibits a slight initial increase before gradually decreasing. At the same mechanical state, *P*^el^ generally increases with increasing *μ*, indicating a higher rate of net elastic energy accumulation under higher interparticle friction. As shearing proceeds, *P*^el^ decreases to zero for all frictional conditions. According to Wang et al. (2024) [40], this condition approximately coincides with the peak stress state, at which the total elastic energy stored in the granular assembly reaches its maximum. Beyond the peak stress, *P*^el^ becomes negative, as shown in Figure 8c, indicating that elastic energy release exceeds elastic energy storage and that part of the elastic energy accumulated during the preceding loading stage is released. This transition accompanies the macroscopic post-peak softening regime, during which continued contact network reorganization reduces the load bearing capacity of the granular assembly. With further shearing, *P*^el^ gradually increases and eventually fluctuates around zero, suggesting that the total stored elastic energy approaches a statistically steady level as the critical state develops.

It is worth noting that the above evolution becomes increasingly pronounced with increasing *μ*. In particular, under higher friction conditions, the transition of *P*^el^ from positive to negative values becomes more significant, accompanied by a larger negative peak. This indicates that higher interparticle friction is associated with greater net elastic energy accumulation before the peak stress and more pronounced elastic energy release during post-peak softening. These results demonstrate that increasing interparticle friction not only modifies the frictional dissipation process but also influences the evolution of elastic energy within the contact network. Accordingly, specimens with higher interparticle friction exhibit higher peak stresses and more pronounced post-peak strain softening.

**Figure 9:**
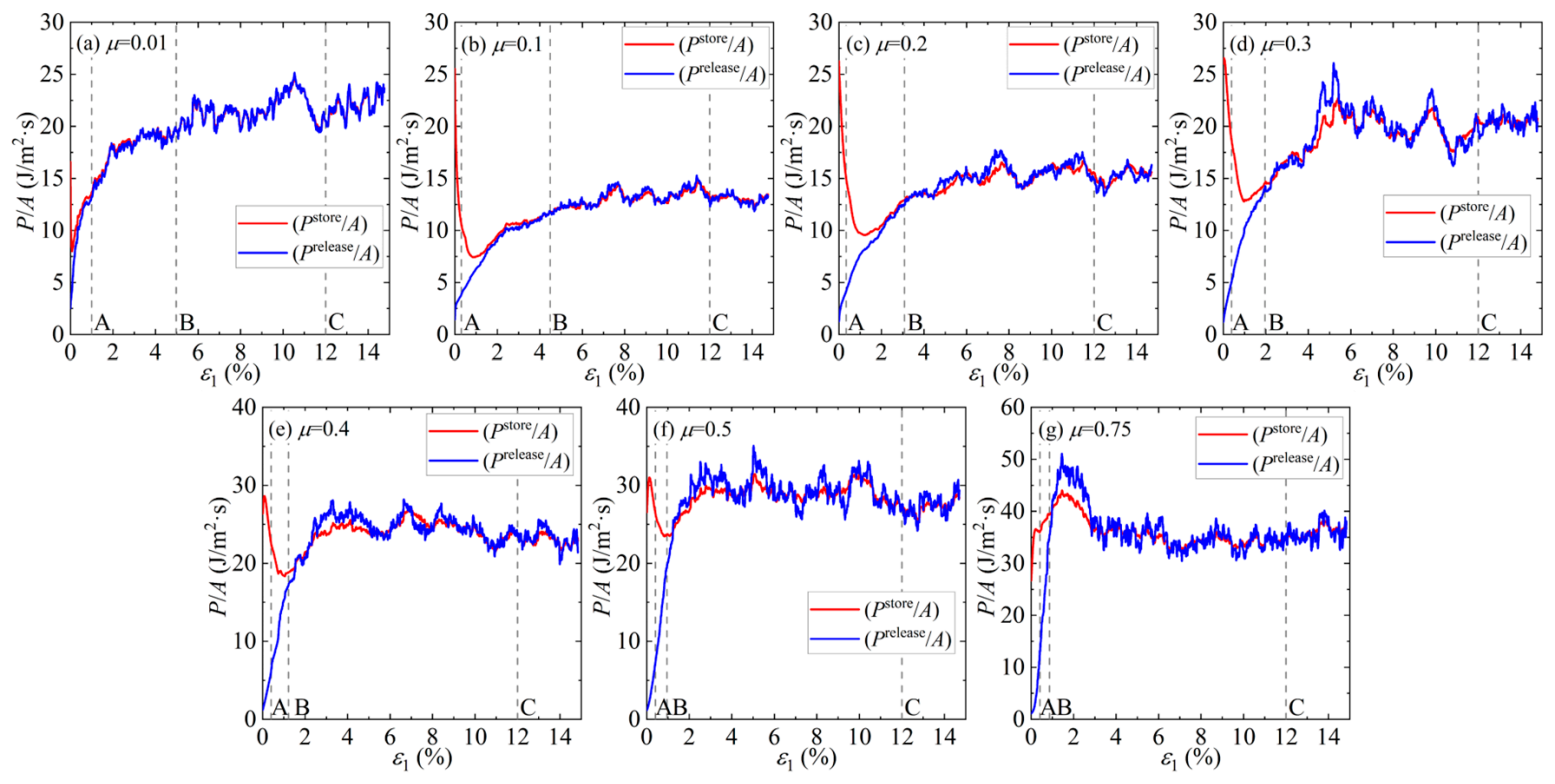
Evolutions of the elastic storage and release powers during shearing under different frictional conditions, normalized by the specimen volume *A*: (**a**) *μ* = 0.01; (**b**) *μ* = 0.1; (**c**) *μ* = 0.2; (**d**) *μ* = 0.3; (**e**) *μ* = 0.4; (**f**) *μ* = 0.5; and (**g**) *μ* = 0.75. The vertical dashed lines indicate three characteristic states: maximum contraction state A, peak stress state B, and critical state C.

To further elucidate the microscopic mechanism governing the evolution of elastic energy in granular assemblies, the variation in elastic energy at each contact $dE_{c}^{\mathrm{el}}$ is examined within an infinitesimal loading increment. A positive value of $dE_{c}^{\mathrm{el}}$ indicates that the contact stores elastic energy during the increment, thereby undergoing a micro-loading process. In contrary, a negative value represents that the contact releases elastic energy, corresponding to a micro-unloading process. By summing the elastic energy increments over all loading contacts and unloading contacts, respectively, the total elastic energy storage and release within each loading increment can be obtained. The corresponding elastic energy storage power *P*^store^ and elastic energy release power *P*^release^ are then defined. Figure 9 shows the evolutions of the normalized storage and release powers under different frictional conditions. Consistent with the previous analysis, both quantities are normalized by the specimen volume, and the original curves are smoothed using a moving average procedure based on adjacent data points.

As shown in Figure 9, the storage power *P*^store^ remains higher than the release power *P*^release^ prior to the peak stress state B for all frictional conditions. This suggests that elastic energy storage dominates during this stage, consistent with the positive elastic power in Figure 8. As shearing proceeds, *P*^release^ gradually increases and approaches to the same value with *P*^store^ near the peak stress state B, corresponding to the zero value of the elastic power in Figure 8. Beyond the peak stress, *P*^release^ becomes larger than *P*^store^, indicating a transition from an energy storage regime to an energy release regime. This dominance of elastic energy release becomes more pronounced with increasing friction coefficient, suggesting that higher interparticle friction enables the granular assembly to release a larger amount of the elastic energy accumulated before the peak stress. With continued shearing, the storage and release powers gradually converge and eventually stabilize at nearly the same non-zero value. This convergence indicates that the net rate of change in the total elastic energy becomes approximately zero, while individual contacts continue to undergo microscopic loading and unloading. Elastic energy released from unloading contacts is therefore accompanied by renewed elastic energy storage at other contacts within the evolving network. The critical state consequently represents a statistically steady elastic energy exchange.

**Figure 10:**
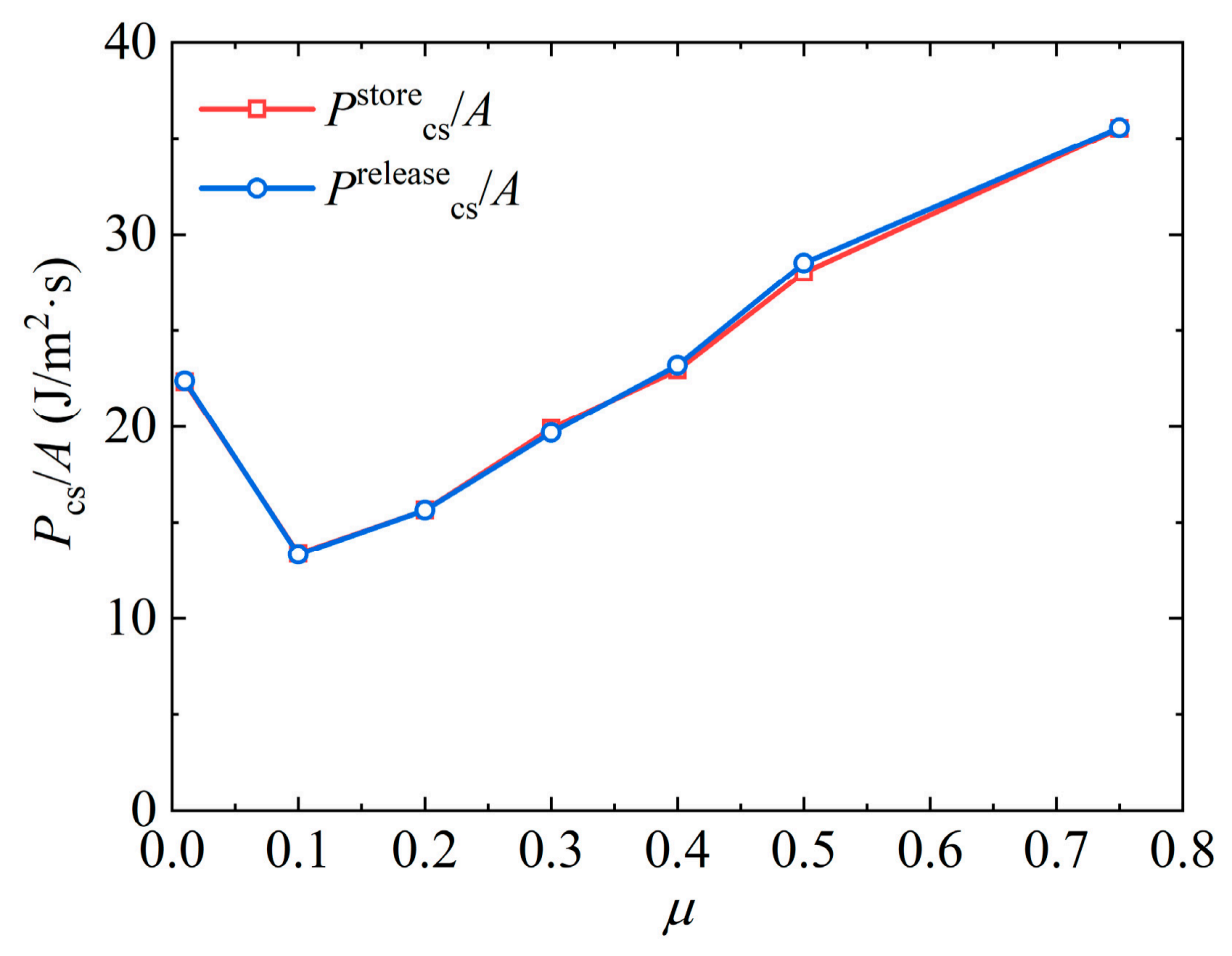
Variations in the critical elastic energy storage and release powers with friction coefficient.

Figure 10 further illustrates the variations in the critical elastic energy storage and release powers with friction coefficient *μ*. The critical values are calculated as the averages over the axial strain range of 12–14%. It can be observed that the storage and release powers exhibit nearly identical trends and display a distinctly non-monotonic dependence on the friction coefficient *μ*. As the friction coefficient increases from 0.1 to 0.75, both the storage and release rates increase approximately linearly, suggesting that although the total elastic energy stored in the system remains essentially constant at the critical state, the microscopic energy exchange within the contact network becomes increasingly active under higher friction conditions. However, under the lowest friction condition (*μ* = 0.01), both the critical storage and release powers are higher than those at *μ* = 0.1. This result suggests that the frequent contact sliding occurring under extremely low friction causes individual contacts to repeatedly experience microscopic loading and unloading, thereby sustaining a high level of elastic energy exchange. Note that the frictional dissipation power at *μ* = 0.01 is lower than that at *μ* = 0.1, suggesting that active topological reconfiguration can still be maintained through the continuous microscopic loading and unloading processes, even though frictional dissipation is reduced. Such structural evolution driven by repeated elastic energy exchange provides a non-dissipative microscopic mechanism for the sustained macroscopic irreversible shear deformation.

### 3.3. Competition Between Dissipative and Non-Dissipative Mechanisms

Considering the continuous storage and release of elastic energy during shearing, the power available to drive particle motion and structural evolution within an incremental loading step consists not only of the externally applied power, but also of the elastic energy released power from microscopic contacts. Accordingly, the total available energy during a loading increment can be expressed as the sum of the external power *P*^ext^ and the elastic release power *P*^release^. This available energy is subsequently partitioned between frictional dissipation and elastic energy storage. Specifically, part of the available power is dissipated through interparticle sliding as frictional dissipation *P*^fric^, whereas the remainder is stored at contacts as elastic energy *P*^store^. Thus, the energy balance can be expressed as

*P*^ext^ + *P*^release^ = *P*^store^ + *P*^fric^ (5)

Following Wang et al. (2024) [40], the elastic ratio *R*^el^ and plastic ratio *R*^pl^ are introduced to quantify the fractions of the available energy associated with elastic energy storage and frictional dissipation, respectively:

(6) $$\begin{array}{l} R^{\mathrm{el}}=\frac{P^{\mathrm{store}}}{P^{\mathrm{ext}}+P^{\mathrm{release}}}\\ R^{\mathrm{pl}}=\frac{P^{\mathrm{fric}}}{P^{\mathrm{ext}}+P^{\mathrm{release}}} \end{array}$$

**Figure 11:**
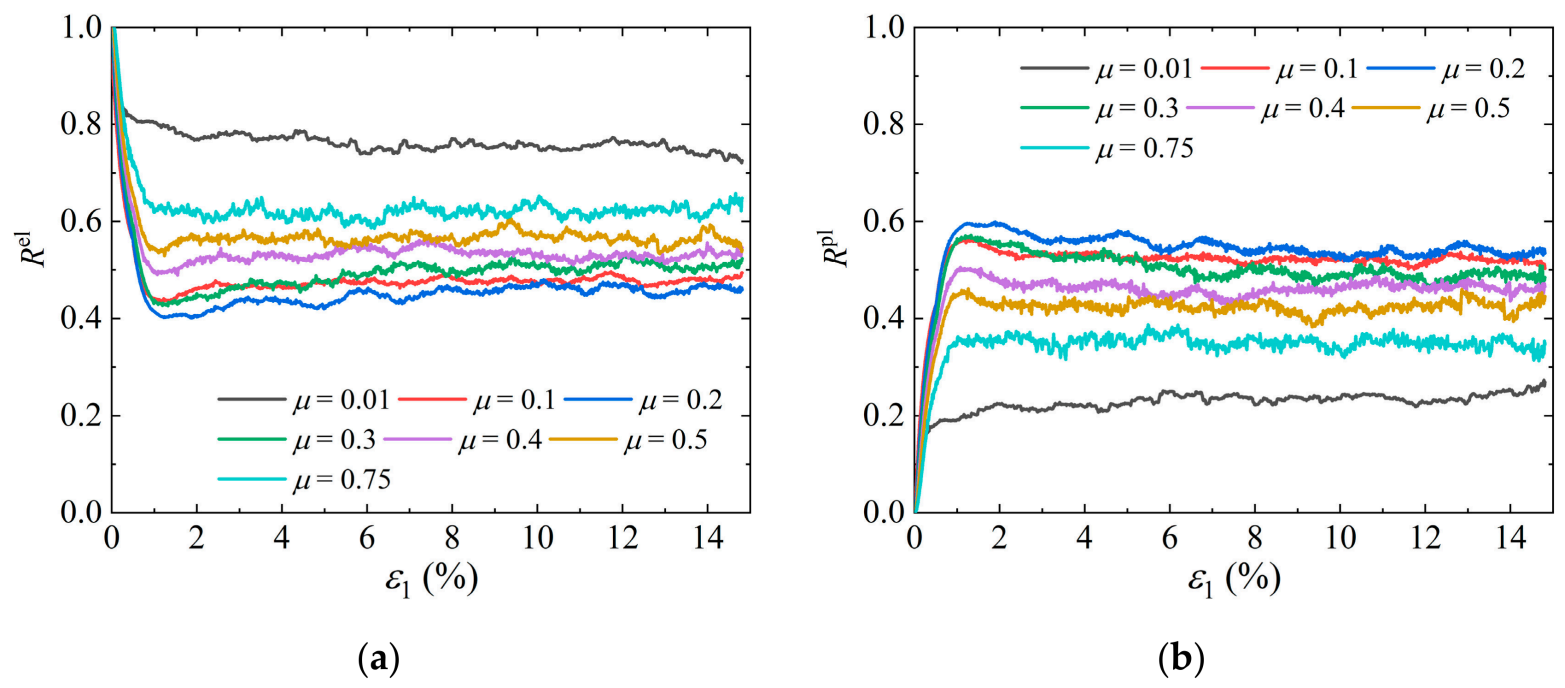
Evolutions of (**a**) the elastic ratio *R*^el^ and (**b**) plastic ratio *R*^pl^ during shearing under different frictional conditions.

Figure 11 presents the evolutions of *R*^el^ and *R*^pl^ during shearing under different frictional conditions. Note that *R*^el^ and *R*^pl^ characterize the non-dissipative energy storage capacity and the dissipative capacity of the granular assembly, respectively. And the two ratios exhibit complementary evolutions throughout the shearing process. As shown in Figure 11, during the initial loading stage, *R*^el^ rapidly decreases from its initial value of 1.0 to a minimum for all frictional conditions, whereas *R*^pl^ increases correspondingly from zero to a maximum. This suggests that an increasing fraction of the available energy is diverted from elastic energy storage to frictional dissipation as shearing proceeds. After that, under both the very low frictional condition (*μ* = 0.01) and the high frictional condition (*μ* = 0.75), *R*^el^ and *R*^pl^ gradually approach stable values. In contrast, when 0.1 ≤ *μ* ≤ 0.5, *R*^el^ (or *R*^pl^) exhibits a slight increase (or decrease) before gradually stabilizing. At the critical state, both ratios approach non-zero, approximately constant values for each frictional condition, suggesting that the energy partitioning within the granular assembly reaches a statistically steady regime. A finite fraction of the available power continues to be stored elastically within the evolving contact network, while the remaining fraction is continuously dissipated through interparticle friction. Therefore, the critical state is characterized by persistent microscopic elastic energy exchange together with a statistically stable partition of the available energy between non-dissipative elastic storage and irreversible frictional dissipation.

**Figure 12:**
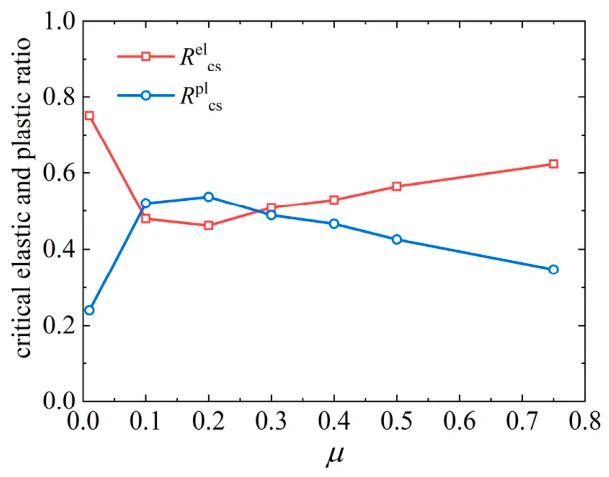
Variations in the critical elastic ratio and plastic ratio with friction coefficient *μ*.

Figure 12 further illustrates the variations in the critical elastic ratio $R_{\mathrm{cs}}^{\mathrm{el}}$ and plastic ratio $R_{\mathrm{cs}}^{\mathrm{pl}}$ with friction coefficient *μ*, where the critical values are considered as the averages over the axial strain range of 12–14%. It can be observed that both ratios exhibit pronounced non-monotonic variations with increasing *μ*. The critical elastic ratio $R_{\mathrm{cs}}^{\mathrm{el}}$ reaches its maximum value of approximately 0.75 at *μ* = 0.01, indicating that a relatively large fraction of the available power is associated with elastic energy storage under the very low-friction condition. This result is consistent with the relatively high elastic energy storage and release powers observed at *μ* = 0.01 in Figure 10. As the friction coefficient increases to 0.1, $R_{\mathrm{cs}}^{\mathrm{el}}$ decreases markedly and reaches its minimum at *μ* = 0.2. Thereafter, the critical elastic ratio $R_{\mathrm{cs}}^{\mathrm{el}}$ increases progressively with increasing *μ*. When ignoring the slight reduction at *μ* = 0.2, the elastic ratio increases approximately linearly for *μ* ≥ 0.1. This suggests that a progressively larger fraction of the available energy is re-stored within the contact network, whereas the fraction consumed by frictional dissipation decreases correspondingly as interparticle friction increases. It is worth noting that, for all cases with *μ* ≥ 0.1, the value of $R_{\mathrm{cs}}^{\mathrm{el}}$ remains within the range of 0.4–0.6. This indicates that, the non-dissipative elastic energy storage mechanism and the frictional dissipation mechanism contribute at comparable levels during continuous shearing at the critical state. In other words, the maintenance of macroscopic irreversible deformation cannot be attributed solely to frictional dissipation, but instead results from the combined action of frictional dissipation and non-dissipative elastic energy exchange.

## 4. Irreversibility of the Non-Dissipative Mechanism

**Figure 13:**
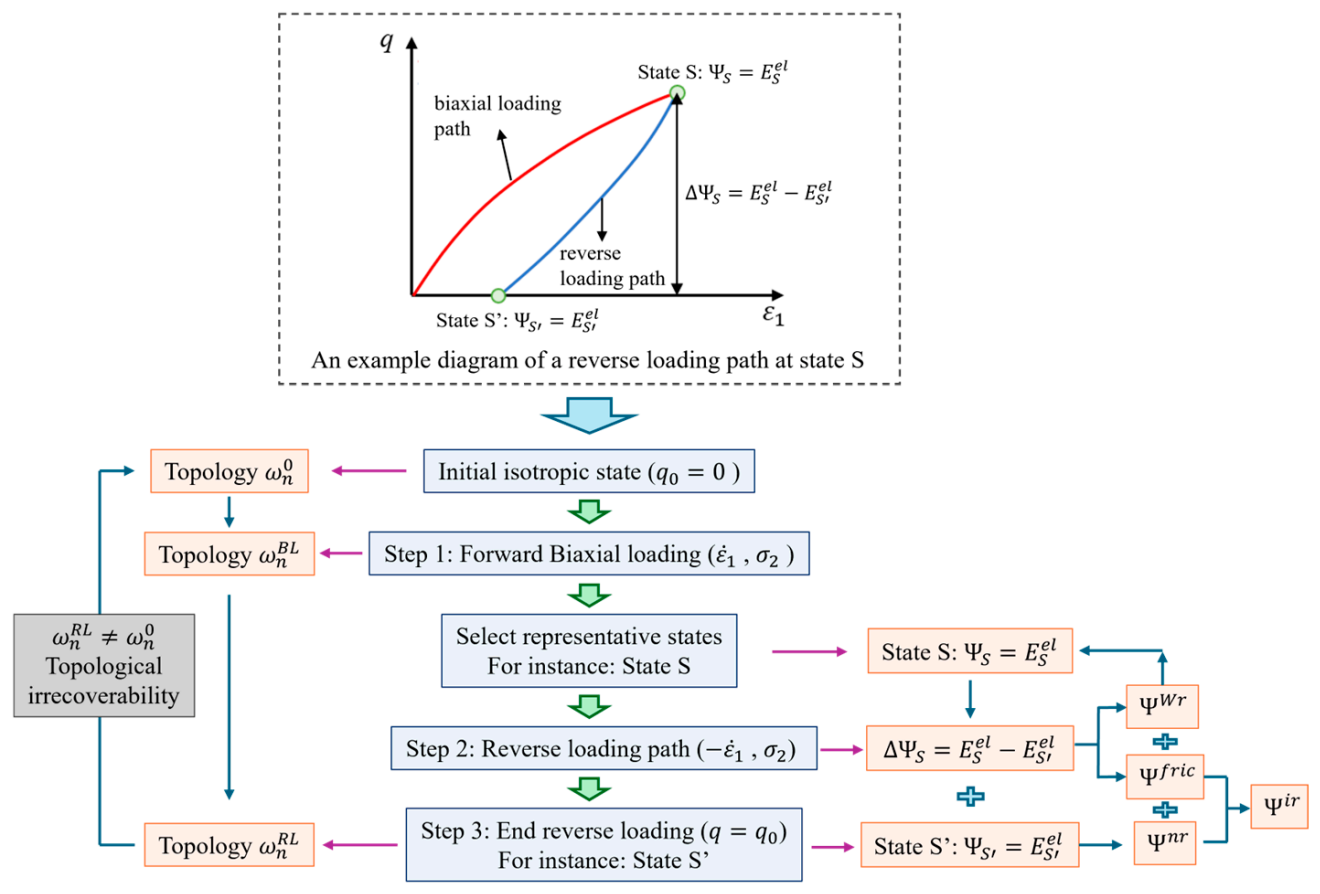
Flowchart of the reverse loading procedure for evaluating topological irrecoverability and free energy partitioning in sheared granular assemblies. The arrows indicate the workflow and associated relationships, while the colors are used for visual distinction only.

To further analyze the irreversibility of the non-dissipative mechanism, a reverse loading procedure is employed. The underlying assumption is that macroscopic stress recovery and microscopic structural recovery are not necessarily equivalent. Therefore, reverse loading is used as a diagnostic procedure to examine whether the contact network topology and the elastic energy stored within the granular assembly can be recovered after the macroscopic stress state is restored. Along the forward biaxial loading path, representative mechanical states are selected at axial strain intervals of 0.1% before the axial strain reached 1%, and subsequently at intervals of 0.5%. For each selected state, reverse axial loading is applied under constant confining pressure at the same loading rate as that used in the forward biaxial loading process (0.01 s^−1^), but in the opposite direction. The reverse loading is terminated when the deviatoric stress returns to its value at the corresponding initial state, as detailed in Liu and Wang (2026) [43]. The detailed computational procedure, stress path, and analysis workflow of the reverse loading are schematically illustrated in Figure 13. This stress recovery criterion defines the endpoint of each reverse loading process, but does not imply complete recovery of the internal contact-network configuration.

The subsequent analysis considers three quantities that characterize different aspects of the recovery process. First, the meso-loop proportions after reverse loading are compared with those of the initial state after isotropic consolidation to quantify the irrecoverability of topological reconfiguration. Second, the residual elastic energy after reverse loading is evaluated to quantify the portion of the stored free energy that remains unrecovered after macroscopic stress recovery. Third, the frictional dissipation generated during reverse loading is evaluated to characterize the irreversible loss associated with the recovery of the recoverable component of free energy.

### 4.1. Irrecoverability of Topological Reconfiguration

**Figure 14:**
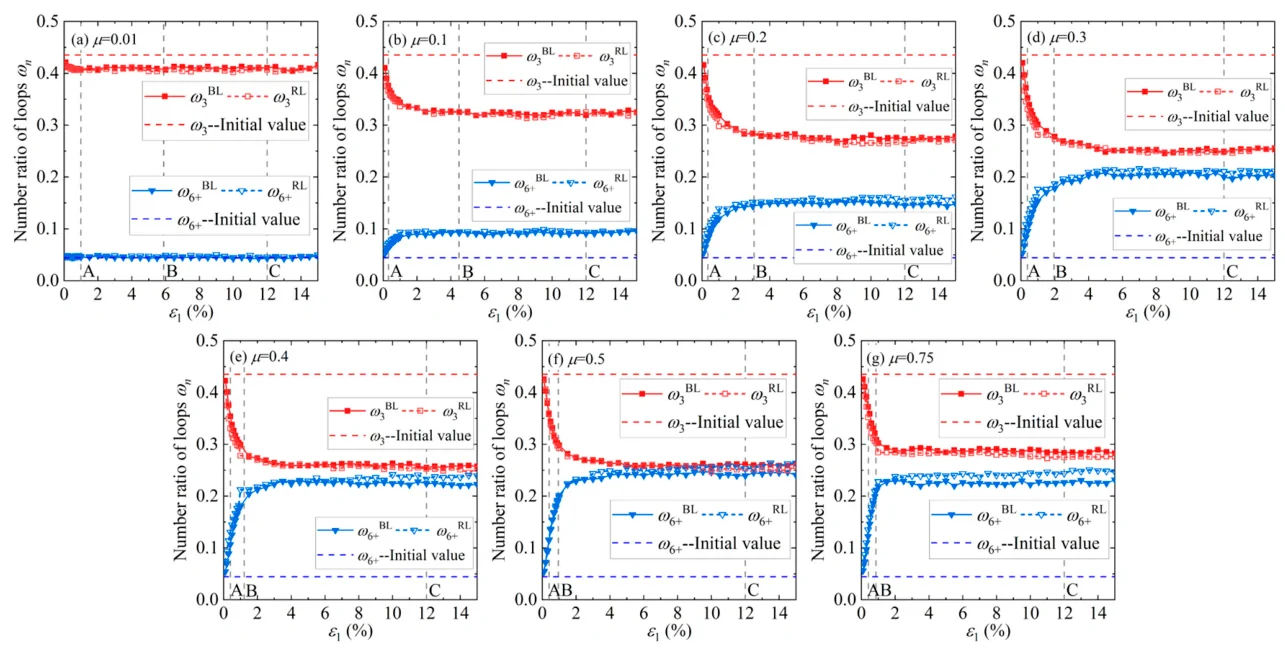
Evolutions of the proportions of lower-order loops L3 and higher-order loops L6+ along the forward biaxial loading path and after the corresponding reverse loading paths in terms of the axial strain corresponding to each selected mechanical state under different frictional conditions: (**a**) *μ* = 0.01; (**b**) *μ* = 0.1; (**c**) *μ* = 0.2; (**d**) *μ* = 0.3; (**e**) *μ* = 0.4; (**f**) *μ* = 0.5; and (**g**) *μ* = 0.75. The vertical dashed lines indicate three characteristic states: maximum contraction state A, peak stress state B, and critical state C.

Figure 14 presents the evolutions of the proportions of lower-order loops L3 and higher-order loops L6+ along the forward biaxial loading path and after the corresponding reverse loading paths under different frictional conditions. The loop proportions are plotted against the axial strain corresponding to each selected mechanical state, and the dashed lines denote the initial topological configuration after isotropic consolidation. The loop proportions along the forward loading path and after reverse loading are denoted by $\omega_{Ln}^{\mathrm{BL}}$ and $\omega_{Ln}^{\mathrm{RL}}$, respectively. As shown in Figure 14, during forward shearing, a continuous topological reconfiguration characterized by the transformation from lower-order to higher-order meso-loops is observed. After the reverse loading process is completed, neither the proportions of L3 loops nor those of L6+ loops recover to their initial values after consolidation. In other words, $\omega_{Ln}^{\mathrm{RL}}$ always deviates from the initial topological state, despite the complete recovery of the macroscopic stress state. The evolution of $\omega_{Ln}^{\mathrm{RL}}$ closely follows that of $\omega_{Ln}^{\mathrm{BL}}$ with only minor quantitative differences. This indicates that, although unloading restores the macroscopic stress to its initial level, the contact network cannot recover its original topology and instead retains the newly formed configuration developed during shearing. Therefore, the topological reconfiguration induced by shearing is intrinsically irrecoverable at the contact network level. Its irrecoverability is associated with contact network reconfiguration and particle rearrangement that persist even after the macroscopic stress state has been restored.

**Figure 15:**
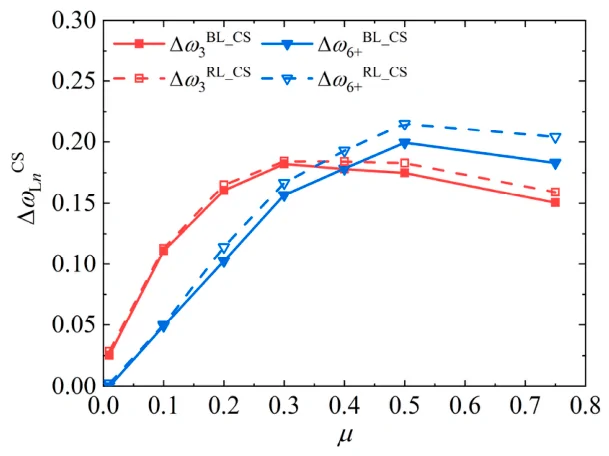
Variations of $\Delta \omega_{Ln}^{\mathrm{CS}}$ for both the forward loading path and the corresponding reverse loading state with *μ*.

To further quantify the irrecoverability of topological reconfiguration relative to the initial state, the difference between the loop proportion at the critical state $\omega_{Ln}^{\mathrm{CS}}$ and its initial value after consolidation $\omega_{Ln}^{0}$ is adopted as an index, i.e., $\Delta \omega_{Ln}^{\mathrm{CS}}=\omega_{Ln}^{\mathrm{CS}}-\omega_{Ln}^{0}$. This index is evaluated for both the forward loading path and the corresponding reverse loading state. The variations of $\Delta \omega_{Ln}^{\mathrm{CS}}$ with *μ* are shown in Figure 15. It can be observed that both L3 and L6+ loops exhibit a pronounced increase in $\Delta \omega_{Ln}^{\mathrm{CS}}$ with increasing *μ* for *μ* < 0.3. Under low friction, tangential sliding at particle contacts is readily activated, promoting continuous contact breakage and formation and thereby facilitating widespread meso-loop reconfiguration. When *μ* increases from 0.3 to 0.5, $\Delta \omega_{Ln}^{\mathrm{CS}}$ of L3 loops decreases, whereas that for L6+ loops gradually increases. This contrasting evolution suggests that increasing friction progressively suppresses relative sliding and particle rearrangement, thereby modifying both the extent and mode of topological reconfiguration. Consequently, beyond the intermediate friction regime, a further increase in frictional resistance does not result in a proportional increase in topological reconfiguration. As *μ* further increases to 0.75, $\Delta \omega_{Ln}^{\mathrm{CS}}$ of both L3 and L6+ loops becomes slightly smaller than that at *μ* = 0.5, suggesting that the suppression of particle rearrangement at very high friction may partially offset the tendency toward further topological transformation. This trend is consistent with the evolution of critical frictional dissipation and macroscopic volumetric strain presented in the previous sections, indicating a close relationship between topological reconfiguration and the macroscopic irreversible deformation of granular assemblies.

### 4.2. Irreversible Free Energy

Based on the first and second laws of thermodynamics, and under the assumption that dissipated energy is transferred to the surroundings as heat under isothermal conditions, the thermodynamic relation for a granular assembly subjected to quasi-static loading, corresponding to the limiting case of zero granular temperature, can be expressed as

δ*W* − δΨ = δΦ (7)

where *W* is the external work input, Ψ is the Helmholtz free energy, and Φ is the dissipated energy. In the present DEM framework, these quantities are directly evaluated from the mechanical and energy responses of the simulated granular assembly. Specifically, the external work *W* is obtained from the mechanical work performed by the moving boundaries during the loading process. The dissipated energy Φ corresponds to the cumulative frictional dissipation energy *E*^fric^ generated by relative tangential sliding at interparticle contacts. For the present quasi-static granular system, the Helmholtz free energy Ψ is represented by the net elastic energy *E*^el^ as described in Section 3.2.

#### 4.2.1. Irrecoverable Free Energy Induced by Topological Reconfiguration

**Figure 16:**
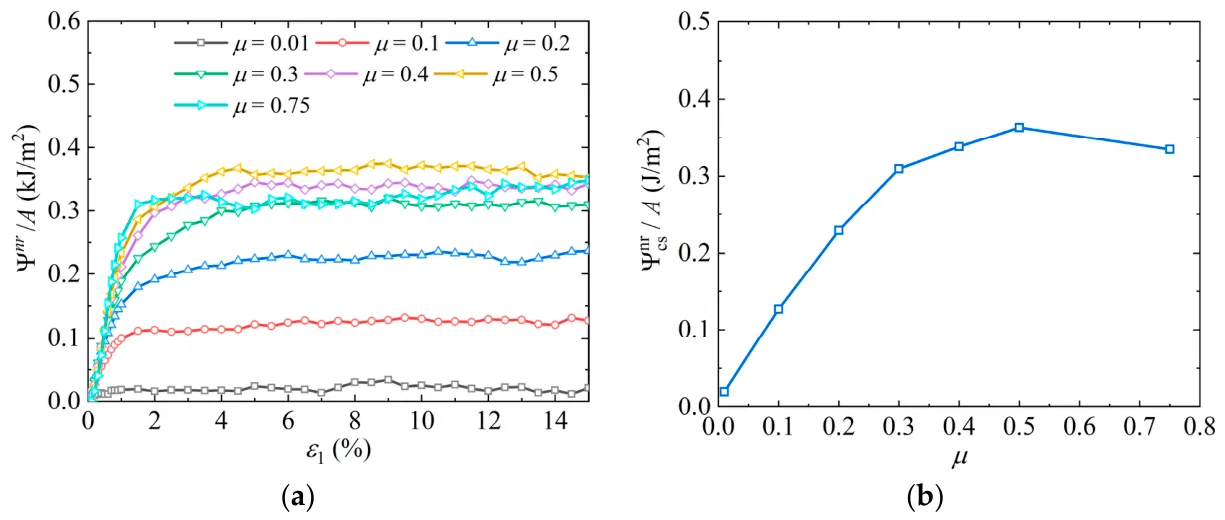
(**a**) Evolution of the non-released free energy with the axial strain corresponding to each selected mechanical state; (**b**) variation in the critical non-released free energy with *μ*.

Since elastic energy is stored locally within the interparticle contact network, the irrecoverability of the topological configuration is expected to directly affect the recoverability of the stored elastic energy. To quantify this effect, the residual elastic energy remaining in the system after reverse loading to the initial deviatoric stress *q* is calculated and denoted by Ψ^nr^. If the stored elastic energy is fully recoverable, all elastic energy would be released upon completion of reverse loading, yielding Ψ^nr^ = 0. However, the evolution of Ψ^nr^ shown in Figure 16a demonstrates that a finite amount of elastic energy consistently remains trapped in the system for all frictional conditions. This suggests that only a fraction of the elastic energy accumulated during forward shearing can be recovered through reverse loading. In this study, this residual component is defined as the irrecoverable free energy. For all frictional conditions, Ψ^nr^ increases continuously during the initial shearing, indicating the progressive storage of irrecoverable free energy within the evolving contact network. As the critical state is approached, Ψ^nr^ gradually reaches an approximately constant plateau. This suggests that irrecoverable free energy is no longer accumulated, which corresponds to the statistical balance between elastic energy storage and release.

The existence of irrecoverable free energy further demonstrates that the irrecoverability of the topological configuration directly results in the loss of elastic energy recoverability. Since elastic energy is stored at interparticle contacts, the topological reconfiguration and associated particle rearrangement developed during shearing can leave a residual elastic energy component after reverse loading. This residual energy therefore provides an energy measure of the microscopic irrecoverability identified from the meso-loop evolution in Section 4.1. In this sense, topological reconfiguration is associated with the retention of part of the externally inputted work within the reconfigured contact network, providing a non-dissipative contribution to the irreversible response of the granular assembly.

Figure 16b further shows the variation in the irrecoverable free energy at the critical state $\Psi_{\mathrm{cs}}^{\mathrm{nr}}$ with friction coefficient *μ*. With the degree of configurational reorganization increasing, $\Psi_{\mathrm{cs}}^{\mathrm{nr}}$ increases progressively with increasing *μ*, while the rate of increase becomes weaker when *μ* > 0.3. Note that the critical irrecoverable free energy at *μ* = 0.75 is slightly lower than that at *μ* = 0.5. This non-monotonic evolution aligns with the trends observed in meso-loop reconfiguration (Figure 15) and critical frictional dissipation power (Figure 5). From the microscopic perspective, increasing friction suppresses relative contact sliding and particle rearrangement, which can change the mode and extent of meso-loop reconfiguration and consequently the amount of elastic energy retained after stress recovery. These results suggest that frictional dissipation and irrecoverable free energy represent two closely related but distinct aspects of macroscopic irreversible deformation. The former arises from dissipative interparticle interactions, whereas the latter is retained within the reconfigured contact network.

#### 4.2.2. Frictional Dissipation of Recoverable Free Energy During Reverse Loading

The free energy stored within the interparticle contact network during forward shearing can be further divided into an irrecoverable component and a recoverable component. The recoverable component can be released during reverse loading, driving the macroscopic recovery of the granular assembly toward its initial stress state. Since reverse loading is performed under quasi-static conditions, the associated energy evolution can be analyzed within the same energy framework described by Equation (7). During reverse loading, part of the released recoverable free energy is converted into frictional dissipation through interparticle sliding Ψ^fric^, while the remaining part is used to recover the macroscopic deformation of the granular assembly Ψ^Wr^. Thus, reverse loading does not represent a purely elastic recovery process. Local particle rearrangements and contact network adjustments continue to occur, causing part of the recoverable free energy to undergo irreversible frictional dissipation. In this sense, the frictional dissipation energy generated from the recoverable free energy component during reverse loading characterizes the irreversible loss of recoverable free energy during the recovery process.

**Figure 17:**
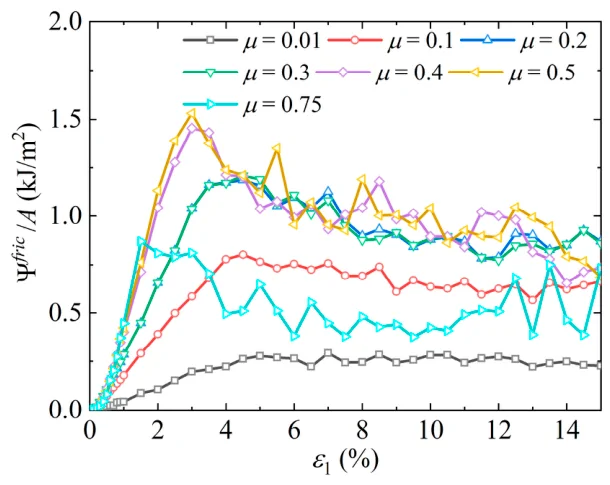
Evolution of the cumulative frictional dissipation energy at the end of reverse loading with the axial strain corresponding to the selected states along the forward loading path under different frictional conditions.

Figure 17 illustrates the evolution of the cumulative frictional dissipation energy Ψ^fric^ at the end of reverse loading as a function of the axial strain corresponding to the selected states along the forward loading path under different frictional conditions. It can be observed that, for all cases of *μ*, Ψ^fric^ initially increases with the axial strain of the selected forward loading state, reaches a maximum value at a certain mechanical state, and subsequently decreases before approaching a stable value near the critical state. This evolution indicates that, during the early stage of reverse loading, the recoverable free energy provides additional energy to activate contact sliding and structural adjustment, resulting in enhanced frictional dissipation. As the system approaches the critical state, the recoverable free energy gradually reaches a stable stage during forward shearing, and consequently the additional dissipation capacity during reverse loading also tends to stabilize. Furthermore, the critical value of Ψ^fric^ increases progressively with increasing *μ*, but becomes insensitive for *μ* > 0.3. Note that, when *μ* = 0.75, both the maximum value of Ψ^fric^ and its stable level at the critical state become significantly lower than those at *μ* = 0.5. This indicates that although higher friction enhances the resistance of individual contacts to sliding, excessive frictional constraints restrict particle rearrangement and contact sliding during reverse loading, thereby reducing the ability of recoverable free energy to be converted into frictional dissipation.

As a conclude, the recovery process of the stored free energy is not purely reversible. The recoverable free energy cannot be completely converted into macroscopic recovery work, but is partially dissipated through frictional mechanisms during reverse loading, leading to a further reduction in macroscopic recoverability. Together with the residual elastic energy identified in Section 4.2.1, the irreversible response of the granular assembly involves two distinct energy processes, i.e., retention of elastic energy associated with irrecoverable topological reconfiguration and frictional dissipation of the recoverable component during reverse loading.

#### 4.2.3. Evolution and Partition of Irreversible Free Energy

To quantify the combined energy effects described above, the irreversible free energy Ψ^ir^ is defined as the sum of the irrecoverable free energy Ψ^nr^ and the frictional dissipation energy generated from the recoverable free energy Ψ^fric^ during the reverse loading process:

Ψ^ir^ = Ψ^nr^ + Ψ^fric^ (8)

This definition provides a measure of the total irreversible energetic contribution identified from the forward and reverse loading processes.

**Figure 18:**
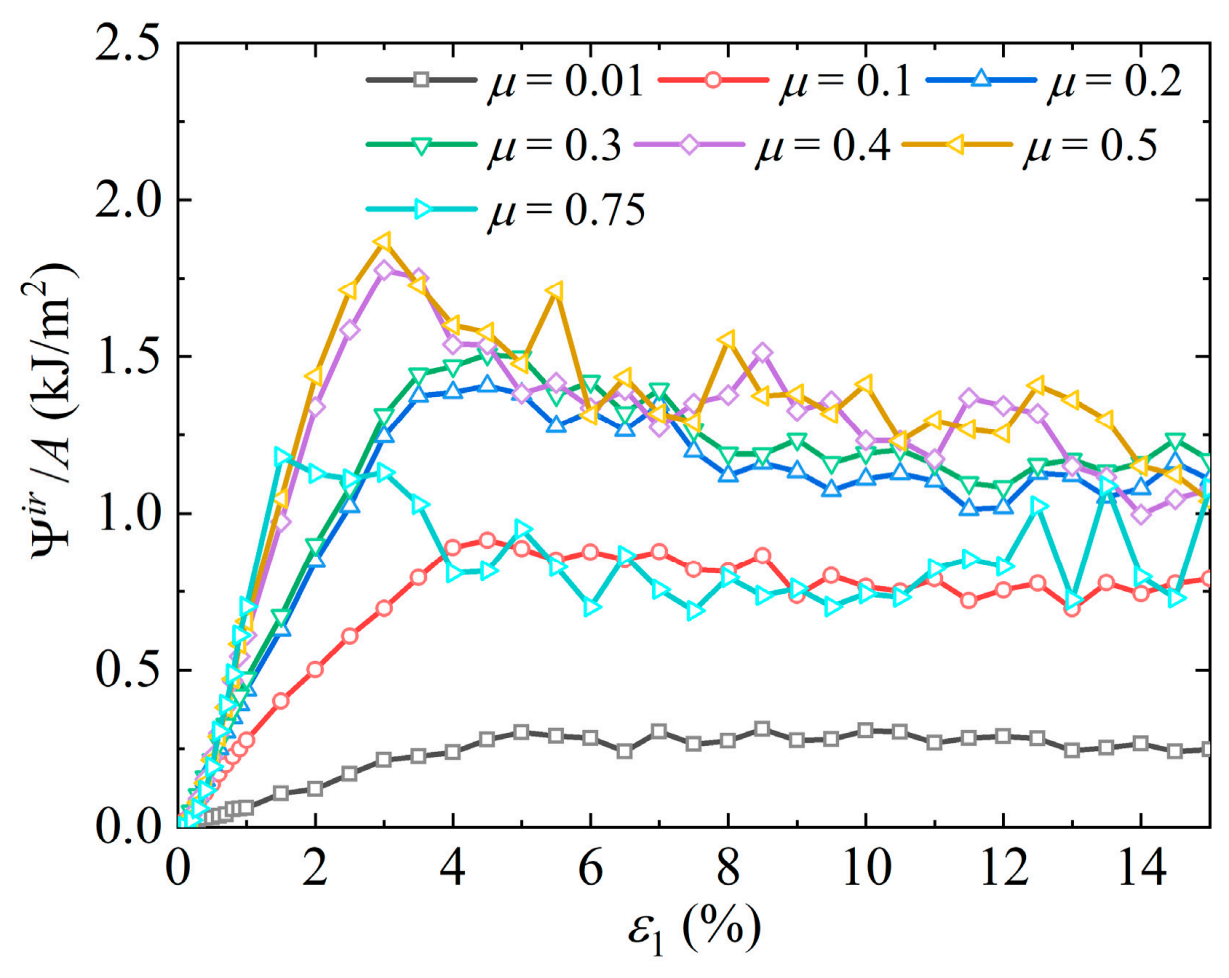
Evolution of the irreversible free energy with the axial strain corresponding to the selected states along the forward loading path under different frictional conditions.

Figure 18 shows the evolution of Ψ^ir^ with axial strain for the selected states along the forward loading path under different frictional conditions. The irreversible free energy initially increases with increasing axial strain, reflecting the progressive development of contact network reconfiguration and the associated accumulation of irrecoverable and frictionally dissipative energy contributions. After reaching a peak value at a certain mechanical state, Ψ^ir^ gradually decreases and eventually approaches a stable level at the critical state. Furthermore, the dependence of Ψ^ir^ at the critical state on the friction coefficient follows a trend similar to that of Ψ^fric^; i.e., it generally increases with increasing friction coefficient and becomes insensitive for *μ* > 0.3, and decreases slightly when *μ* increases from 0.5 to 0.75. The existence of irreversible free energy indicates that macroscopic irreversible deformation in granular assemblies is associated not only with conventional frictional dissipation but also with the irreversible evolution of free energy associated with microscopic topological reconfiguration.

**Figure 19:**
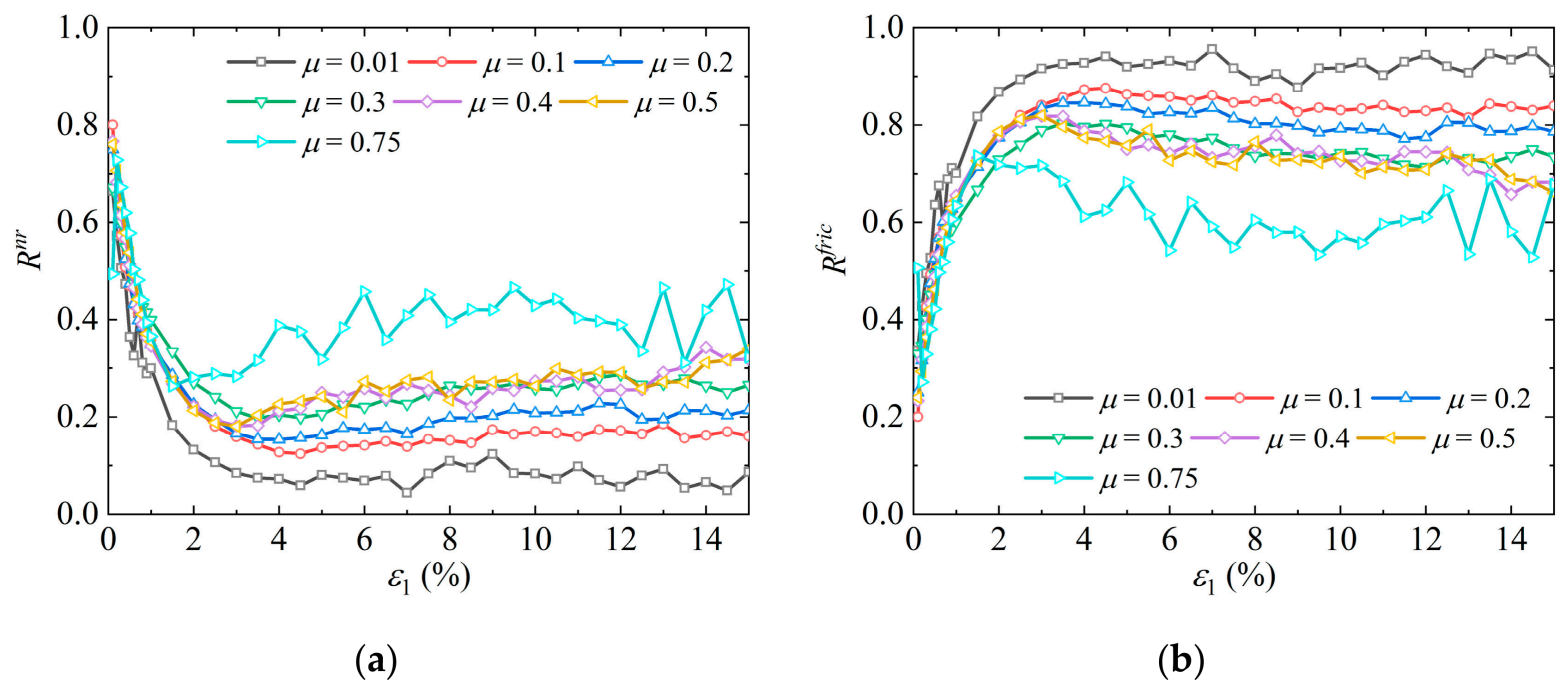
Evolution of (**a**) *R*^nr^ and (**b**) *R*^fric^ with the axial strain corresponding to the selected states along the forward loading path under different frictional conditions.

The contributions of Ψ^nr^ and Ψ^fric^ to the total irreversible free energy Ψ^ir^ are further quantified by defining their respective ratios *R*^nr^ and *R*^fric^. Figure 19 shows the evolution of *R*^nr^ and *R*^fric^ with the axial strain corresponding to the selected forward loading states. For all different frictional conditions, *R*^nr^ initially decreases from a relatively high value greater than 0.5, reaches a minimum value, and then slightly increases toward an approximately stable value near the critical state. Correspondingly, *R*^fric^ exhibits the complementary evolution. This suggests that, during the initial loading stage, significant topological reconfiguration of the granular assembly results in the majority of irreversible free energy being stored in the form of irrecoverable free energy at the contacts. Therefore, the non-dissipative mechanism dominates the irreversible evolution of free energy at this stage. As shearing proceeds, a more stable contact structure gradually develops within the granular assembly. In this sense, an increasing proportion of newly stored free energy can be released during reverse loading and subsequently converted into frictional dissipation.

In addition, the value of *R*^nr^ at the critical state increases with increasing friction coefficient, whereas *R*^fric^ exhibits the opposite trend. This indicates that stronger frictional constraints are associated with a larger relative contribution of irrecoverable elastic energy to the total irreversible energetic measure. Nevertheless, *R*^fric^ remains below 0.4 under all frictional conditions investigated. Thus, within the present definition of irreversible free energy, the irrecoverable component does not account for the majority of the irreversible energetic contribution at the critical state; frictional dissipation associated with the recoverable component remains an essential contribution.

## 5. Conclusions

Based on a series of DEM biaxial simulations with different interparticle friction coefficients, this study investigates the microscopic mechanisms of irreversibility in sheared granular assemblies. The topological reconfiguration and the associated elastic energy evolution of granular assemblies during shearing is analyzed. And the irreversibility of the non-dissipative mechanism within the granular assemblies is further discussed according to the reverse loading results. The main conclusions are summarized as follows.

(1) Granular assemblies undergo persistent topological reconfiguration during shearing through the continuous generation and disappearance of meso-loops, eventually reaching a topological steady state at the critical state. The associated elastic energy storage and release at the contact level constitute a non-dissipative energy mechanism within the evolving contact network. At the critical state, storage and release powers remain finite and statistically balanced, demonstrating that microscopic dynamic energy exchange persists alongside primary frictional dissipation even when macroscopic stress and volume remain stationary.
(2) When reverse loading restores the deviatoric stress to its initial value, the meso-loop topology retains a permanent residual reconfiguration. This irrecoverability is fundamentally quantified by the residual contact strain energy locked within the contact network structure. The topological irrecoverability exhibits a non-monotonic dependence on the friction coefficient, following the same trend as the critical frictional dissipation and macroscopic volumetric strain. This suggests a close relationship between topological reconfiguration and the macroscopic irreversible deformation of granular assemblies.
(3) The free energy accumulated during forward shearing is only partially recoverable upon unloading. Topological reconfiguration irreversibly locks part of the externally input work within the reconstructed contact network, forming irrecoverable free energy that represents the microscopic irreversibility of the non-dissipative mechanism. The remaining recoverable free energy is partly dissipated through friction during unloading, leading to a discrepancy between microscopic and macroscopic recoverability.
(4) By combining the residual elastic energy associated with irrecoverable topological reconfiguration and the frictional dissipation generated from the recoverable component during unloading, the irreversible free energy characterizes the non-dissipative mechanism contributing to the macroscopic irreversible deformation of granular assemblies. Its variation with interparticle friction is consistent with that of frictional dissipation at the critical state, indicating that macroscopic volumetric deformation is jointly governed by dissipative and non-dissipative mechanisms.

The present study identifies contact network topological reconfiguration as a non-dissipative microscopic mechanism and establishes its energy consequence through irrecoverable free energy. The key implication is that macroscopic stress recovery does not necessarily correspond to recovery of the microscopic structural and energy state. This distinction provides a new perspective for understanding path dependence and hysteresis in granular materials, particularly during unloading–reloading processes. From an application perspective, the proposed irreversible free energy may provide a basis for developing constitutive descriptions in which contact network topology is introduced together with conventional frictional dissipation to represent irreversible deformation. It may also provide a microscopic interpretation for residual deformation and hysteresis observed in geotechnical loading–unloading processes. It should be noted that the present study is restricted to two-dimensional DEM simulations with idealized circular particles and a linear contact model. The 2D representation inevitably simplifies the three-dimensional contact topology and particle kinematics. Therefore, the present results should be interpreted as a mechanistic analysis of topological reconfiguration. Future work should extend to three-dimensional granular systems to investigate the influence of particle shape and particle breakage on the proposed interpretation of irreversible free energy.

## Figures and Tables

**Table 1:** Axial strains corresponding to the three characteristic states under different friction coefficients.

	*μ* = 0.01	*μ* = 0.1	*μ* = 0.2	*μ* = 0.3	*μ* = 0.4	*μ* = 0.5	*μ* = 0.75
State A	0.99%	0.31%	0.34%	0.38%	0.40%	0.41%	0.41%
State B	4.97%	4.49%	3.08%	1.96%	1.22%	0.95%	0.87%
State C	12%	12%	12%	12%	12%	12%	12%

## Data Availability

The original contributions presented in this study are included in the article. Further inquiries can be directed to the corresponding author.
